# Antibiotic Cycling Affects Resistance Evolution Independently of Collateral Sensitivity

**DOI:** 10.1093/molbev/msac257

**Published:** 2022-12-08

**Authors:** Pauline Brepoels, Kenny Appermans, Camilo Andres Pérez-Romero, Bram Lories, Kathleen Marchal, Hans P Steenackers

**Affiliations:** Department of Microbial and Molecular Systems, Centre of Microbial and Plant Genetics (CMPG), KU Leuven, Leuven, Belgium; Department of Microbial and Molecular Systems, Centre of Microbial and Plant Genetics (CMPG), KU Leuven, Leuven, Belgium; Department of Information Technology and the Department of Plant Biotechnology, Biochemistry and Bioinformatics, Ghent University, Ghent, Belgium; Department of Microbial and Molecular Systems, Centre of Microbial and Plant Genetics (CMPG), KU Leuven, Leuven, Belgium; Department of Information Technology and the Department of Plant Biotechnology, Biochemistry and Bioinformatics, Ghent University, Ghent, Belgium; Department of Microbial and Molecular Systems, Centre of Microbial and Plant Genetics (CMPG), KU Leuven, Leuven, Belgium

## Abstract

Antibiotic cycling has been proposed as a promising approach to slow down resistance evolution against currently employed antibiotics. It remains unclear, however, to which extent the decreased resistance evolution is the result of collateral sensitivity, an evolutionary trade-off where resistance to one antibiotic enhances the sensitivity to the second, or due to additional effects of the evolved genetic background, in which mutations accumulated during treatment with a first antibiotic alter the emergence and spread of resistance against a second antibiotic via other mechanisms. Also, the influence of antibiotic exposure patterns on the outcome of drug cycling is unknown. Here, we systematically assessed the effects of the evolved genetic background by focusing on the first switch between two antibiotics against *Salmonella* Typhimurium, with cefotaxime fixed as the first and a broad variety of other drugs as the second antibiotic. By normalizing the antibiotic concentrations to eliminate the effects of collateral sensitivity, we demonstrated a clear contribution of the evolved genetic background beyond collateral sensitivity, which either enhanced or reduced the adaptive potential depending on the specific drug combination. We further demonstrated that the gradient strength with which cefotaxime was applied affected both cefotaxime resistance evolution and adaptation to second antibiotics, an effect that was associated with higher levels of clonal interference and reduced cost of resistance in populations evolved under weaker cefotaxime gradients. Overall, our work highlights that drug cycling can affect resistance evolution independently of collateral sensitivity, in a manner that is contingent on the antibiotic exposure pattern.

## Introduction

The overuse and misuse of antibiotics during the last decades were accompanied by a fast spread of (multi-)resistant pathogens. In 2019, it was estimated that about 4.95 million deaths were associated with antimicrobial resistance among which 1.27 million were directly attributable to antimicrobial resistance (Murray et al. 2022). These numbers are expected to increase to 10 million deaths annually by 2050 (O’Neill 2016; Collins 2018). Resistant bacteria can arise *de novo* even within the course of a single chronic infection, and multidrug resistant bacteria can easily gain additional resistance (Musher et al. 2002; Lieberman et al. 2011; Stone et al. 2011). The development of novel strategies that slow the spread of resistance is therefore crucial. The most straightforward approaches are those that employ drugs that are currently already available.

One possible approach recently gaining popularity is the rational cycling between different antibiotics based on the concept of collateral sensitivity. Collateral sensitivity occurs when resistance against a first antibiotic comes with the trade-off of enhancing the sensitivity to a second antibiotic (Imamovic and Sommer 2013; Lázár et al. 2013, 2014; Oz et al. 2014; Yen and Papin 2017; Yoshida et al. 2017; Imamovic et al. 2018). This enhanced sensitivity during treatment with the second antibiotic can in principle lead to an enhanced extinction of the bacterial population, by reducing the pool of surviving cells from which resistant mutants can emerge or by rendering the effect size of resistance mutations insufficient for them to spread. Alternatively, if only a subpopulation acquired resistance to the first antibiotic and thus collateral sensitivity to the second, this can result in a counter-selection of resistance to the first antibiotic and therefore reduced multidrug resistance (Imamovic and Sommer 2013; Barbosa et al. 2019). Collateral sensitivity has been widely found in laboratory and clinical strains of many species and extensive networks of collateral sensitivity/cross-resistance between antibiotics have been established (Lázár et al. 2013; Kim et al. 2014; Lázár et al. 2014; Oz et al. 2014; Barbosa et al. 2017; Jahn et al. 2017). It is well-recognized that the utility of these networks for medical applications is strongly dependent on their repeatability within and across conditions. Therefore, several studies have focused on comparing collateral sensitivity evolution among parallel bacterial populations (i) exposed to the same treatment (Oz et al. 2014; Barbosa et al. 2017; Nichol et al. 2019) or (ii) subjected to different antibiotic exposure patterns (e.g., strong vs. weak antibiotic gradients) or experimental conditions (e.g., population size) (Oz et al. 2014; Jahn et al. 2017), whereas other studies (iii) evaluated the stability of collateral sensitivity over time (Yen and Papin 2017; Yoshida et al. 2017).

A number of recent studies have put the strategy into practice and followed microbial adaptation in evolution experiments where antibiotic pairs were cycled once or multiple times at short (e.g., daily) or longer time intervals (Imamovic and Sommer 2013; Kim et al. 2014; Yen and Papin 2017; Yoshida et al. 2017; Imamovic et al. 2018; Barbosa et al. 2019). These experiments yielded promising results and showed high extinction rates and low rates of (multi)drug resistance evolution for specific drug combinations, and a clear dependency of the resistance-associated mutational patterns on drug types and order. It is currently unclear, however, to which extent these evolutionary outcomes are solely dependent on collateral sensitivity effects of resistance mutations to the first antibiotic and their subsequent impact on the emergence and spread of resistance mutations against the second antibiotic. Alternatively, mutations accumulated in the genetic background during exposure to the first antibiotic (including but not limited to those encoding collateral sensitivity) might alter the emergence and spread of resistance mutations against second antibiotics in additional ways. Knowledge of such additional effects of the genetic background on the evolutionary potential is of critical importance to assess the value of collateral sensitivity networks for designing drug cycling strategies since these networks merely summarize the effects of collateral sensitivity. A systematic evaluation of the importance of effects of the genetic background that go beyond collateral sensitivity requires a combination of elements not included in prior evolution experiments: (i) eliminating differences in the effect size of collateral sensitivity by normalization of the antibiotic doses during the second treatment, (ii) direct comparison of resistance evolution against the second drug between populations adapted to the first drug and nonadapted populations, and (iii) inclusion of a sufficient number of parallel replicates to assess repeatability for the given condition. Finally, as for collateral sensitivity, it is important to assess to which extent the effects might be dependent on the applied drug exposure patterns and selective pressures.

Here, we aimed to systematically assess the impact of the effects of the genetic background on resistance evolution during drug cycling that go beyond the effects of collateral sensitivity, by focusing on the first switch between two antibiotics, with the β-lactam cefotaxime fixed as the first antibiotic and a broad variety of other drugs as the second antibiotic. We first exposed five parallel populations of the model pathogen *Salmonella* Typhimurium to a linear gradient of cefotaxime to evolve resistance and assessed collateral sensitivity/cross-resistance against the second antibiotic. Next, we normalized the applied concentration of the second antibiotic to eliminate the effects of collateral sensitivity or cross-resistance and compared the extinction rates with populations that were not preadapted to cefotaxime, but only to the growth medium. Our focus on extinction rate as an evolutionary outcome is motivated by its high relevance for clinical treatment and the possibility of high parallel experiments (500 repeats per antibiotic included in total). In order to elucidate the potential effects of the antibiotic exposure pattern, we repeated the whole analysis for populations that were exposed to four additional, steeper cefotaxime gradients. To further substantiate the impact of the genetic background on the evolutionary potential, we finally explored differences in mutational patterns upon exposure to different cefotaxime gradients and correlated these to cefotaxime resistance levels, costs of resistance, and ultimately extinction rates to second antibiotics.

## Results

### Influence of Preadaptation to Cefotaxime on Resistance Evolution to Second Antibiotics

#### Adaptation to Linearly Increasing Cefotaxime Concentrations Results in High Resistance Levels

**Fig. 1.**
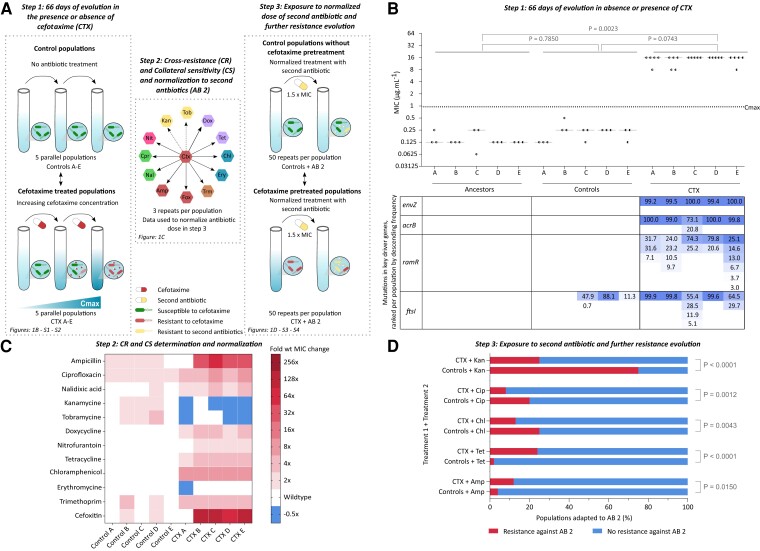
Cefotaxime exposure for 66 days results in a strong increase in cefotaxime resistance and impacts potential to adapt to second antibiotics. (*A*) Experimental setup—Step 1: Five ancestral populations (ancestors A–E) were each transferred for 66 days in the absence (controls A–E) or presence of linearly increasing cefotaxime concentrations (CTX A–E) until a maximum concentration (*C*_max_) of 4 × MIC_A_ (0.95 µg/mL) was reached. Next, the cross-resistance (CR) and collateral sensitivity (CS) levels against 12 additional antibiotics were determined using a MIC assay in step 2. These MIC values were used to normalize (1.5 × MIC) the applied antibiotic concentration in the next step. Finally, in step 3, populations previously evolved in either the absence or presence of cefotaxime were evolved (1.5 × MIC from step 2) against five second antibiotics (AB 2). (*B*) (Top) Populations evolved in the presence of cefotaxime showed elevated cefotaxime resistance levels (µg/mL) compared with the controls and ancestors. Ancestral clones (A–E) represent clones from which the five parallel lineages were initiated. Controls (A–E) were transferred for 66 days in absence of cefotaxime and populations (CTX A–E) were exposed for 66 days to linearly increasing cefotaxime concentrations. Each dot represents the MIC of one independent biological repeat. Horizontal lines represent the median MIC of at least three repeats (*n* ≥ 3). Dashed line indicates the maximum cefotaxime concentration (*C*_max_) at day 66 (4 × MIC_A_ or 0.95 µg/mL). *P*-values were derived from the nonparametric Kruskal–Wallis test followed by a post hoc Dunn's multiple comparison test. (Bottom) Cefotaxime resistance-conferring mutations were mainly present in high frequencies in four-key driver genes of antibiotic resistance. The frequencies of the mutated variants are indicated in descending order per population and per gene; frequencies in one row do not necessarily represent the same variant. (*C*) Heatmap showing resistance levels against 12 antibiotics of populations evolved in the absence (controls A–E) or presence of cefotaxime (CTX A–E). Cross-resistance (*P* < 0.0001) and/or collateral sensitivity (*P* = 0.0233) is more commonly observed in cefotaxime pretreated populations. Color scales indicate fold increase (cross-resistance) or decrease (collateral sensitivity) in antibiotic resistance relative to an untreated ancestral *S.* Typhimurium. *P*-values are derived from the Fischer's Exact test. (*D*) Preadaptation to cefotaxime impacts the potential to adapt to second antibiotics even after normalization (1.5 × MIC) of the antibiotic dose. Populations that were preadapted to cefotaxime show an elevated adaptation compared with the controls when exposed to ampicillin and tetracycline. In contrast, the controls show more adaptation when treated with chloramphenicol, ciprofloxacin, and kanamycin. The percentages of populations able to adapt to the second antibiotic are shown; cefotaxime (CTX), ampicillin (Amp), tetracycline (Tet), chloramphenicol (Chl), ciprofloxacin (Cip), and kanamycin (Kan). *P*-values were derived from a Fisher's Exact test. These data were analyzed after exclusion of hypermutator strains. However, hypermutator strains did not have an effect on the conclusions (supplementary fig. S4, Supplementary Material online). Source data underlying these figures can be found in supplementary tables S4–S6, Supplementary Material online.

In order to unravel the role of exposure to a first antibiotic in resistance evolution against second antibiotics, the common enteropathogen *S.* Typhimurium (EFSA and ECDC 2018; Wang et al. 2019) was first evolved in the presence of the clinically relevant β-lactam antibiotic cefotaxime (EFSA and ECDC 2018). Hereto, five parallel *S.* Typhimurium ATCC14028 lineages (CTX A–E) were serially passaged for 66 days in the presence of linearly increasing cefotaxime concentrations until the populations reached a maximum concentration (*C*_max_) of four times the ancestral minimal inhibitory concentration (4 × MIC_A_ or 0.95 µg/mL) (supplementary fig. S1 and table S1, Supplementary Material online). This concentration is often used in clinical treatment since β-lactam antibiotics show time-dependent killing characteristics, that is the time above the MIC is more important than the applied antibiotic concentration. Moreover, several studies stated that the time above 4 × MIC is an important target in achieving positive treatment outcomes in critically ill patients (Tam 2002; Drusano 2004; Sime et al. 2012; Osthoff et al. 2016; Carrié et al. 2018). In parallel to the cefotaxime-treated populations, five parallel populations were transferred for 66 days without cefotaxime to control for adaptations to culturing conditions (controls A–E) (fig. 1*A*).

After 66 days of evolution either in the presence or absence of cefotaxime, resistance levels at the population level were measured using a MIC assay (fig. 1*B*). The median resistance levels of the ancestors, based on a minimum of three repeats per population, ranged between 0.125 and 0.250 µg/mL. The populations that evolved for 66 days in the presence of cefotaxime showed a strong increase in resistance compared with the ancestors. Moreover, the median resistance level (16 µg/mL) of all cefotaxime-treated populations combined was more than 16 times higher than *C*_max_, indicating a strong overshoot in cefotaxime resistance (fig. 1*B*). Populations able to survive and replicate in higher antibiotic concentrations than those they have adapted to have also been observed in previous evolution experiments with *Escherichia coli* (Imamovic and Sommer 2013; Jahn et al. 2017) and *Pseudomonas aeruginosa* (Imamovic et al. 2018). In contrast, the resistance levels of the controls, which were evolved for 66 days in absence of cefotaxime, only minimally increased to at most double of the ancestor's MIC (fig. 1*B*). Similar minor increases in resistance during experimental evolution without antibiotic exposure have previously been observed for *Salmonella* (Knöppel et al. 2017). Overall, the median resistance levels of the five lineages evolved under the same specific condition were similar, supporting the reproducibility of our experimental setup. Moreover, the MIC levels of 10 randomly selected colonies isolated from each cefotaxime-adapted population were similar to the MIC levels at the population level suggesting that the cefotaxime resistance-conferring mutations are present in high frequencies (supplementary fig. S2 and table S2, Supplementary Material online).

#### Mutations are Mainly Found in Four Antibiotic Resistance-Related Genes

Populations were whole genome sequenced to identify the mutations contributing to the increased resistance levels. To exclude mutations initially present in the populations, the five ancestors were also sequenced. In total, we found 4,288 mutations over all 15 populations compared with the reference strain *S*. Typhimurium [NC_016856.1] (for additional information see Materials and Methods and supplementary table WGS, Supplementary Material online). In order to more easily identify the adaptive mutations, synonymous mutations, mutations occurring in <10% of all sequenced populations, and mutations initially present in the ancestors were removed from further analysis. After this filtering step, a total of 155 mutations were retained, of which 96 in all the controls together and 59 in all the cefotaxime-treated lineages combined (supplementary table S3, Supplementary Material online). The difference between the controls and cefotaxime-treated populations can be attributed to the presence of two hypermutator populations in the controls (control B and control D). 72% of the cells in control D had a mutation in *mutS* (Pro472f), which is known to cause a hypermutator phenotype (Barrick and Lenski 2013; Sheng et al. 2020). In addition, control B also developed hypermutators, since it was for 30% mutated in *mutL* (deletion of Leu72 and Ala73) (Barrick and Lenski 2013; Sheng et al. 2020) (supplementary table S4, Supplementary Material online). Exclusion of these hypermutator populations resulted in an on average similar number of mutations per population in controls and cefotaxime-treated populations (on average 11 and 11.8, respectively) (supplementary tables S3 and S4, Supplementary Material online).

Four genes well-known to contribute to cefotaxime resistance were found to be repeatedly mutated across all cefotaxime-exposed populations, namely *envZ*, *acrB*, *ramR*, and *ftsI* (Lázár et al. 2014; Oz et al. 2014; Sun et al. 2014; Adler et al. 2016; Trampari et al. 2021) (fig. 1*B* ; supplementary table S4, Supplementary Material online). Mutations in these genes accounted for 37 out of 59 mutations in the cefotaxime-exposed populations (supplementary table S3, Supplementary Material online). *envZ* mutations reached fixation in every cefotaxime-exposed population (fig. 1*B*–supplementary table S4, Supplementary Material online). This gene codes for the sensor histidine kinase of the two-component system EnvZ/OmpR that coordinates the expression of the outer membrane proteins OmpF and OmpC (Khorchid et al. 2005; Sun et al. 2009). Mutations in *envZ* are therefore known to influence membrane permeability during treatment with β-lactam antibiotics (Sun et al. 2009; Adler et al. 2013, 2016). Specifically, mutations were found in the second transmembrane domain (EnvZ_163–179_) and the cytoplasmic domain (EnvZ_223–450_) of EnvZ, responsible for signal transduction and kinase activity, respectively (Tanaka et al. 1998; Inouye et al. 1999; Khorchid et al. 2005; Kishii et al. 2007). *EnvZ* mutations in the cytoplasmic domain were previously reported in *Salmonella* after cefotaxime exposure, further supporting that these mutations contribute to cefotaxime resistance (Trampari et al. 2021). The second gene that was mutated across all populations is *acrB*, coding for a multidrug efflux RND transporter permease subunit which is part of the multidrug efflux pump AcrAB/TolC, known to export β-lactam antibiotics (Vargiu and Nikaido 2012; Yamaguchi et al. 2015). *AcrB* mutations were fixed in four out of five cefotaxime-adapted populations (fig. 1*B*; supplementary table S4, Supplementary Material online). Four out of six mutations were found in the periplasmic region of AcrB, which is responsible for substrate translocation and interacts with AcrA (Baucheron et al. 2004; Yamaguchi et al. 2015). Mutations in the periplasmic region of *acrB* have also been observed previously after repeated treatment with cefotaxime (Trampari et al. 2021). The third mutated gene is *ramR,* a TetR family transcriptional regulator repressing the expression of *ramA*, which in turn regulates the expression of *acrAB* (Abouzeed et al. 2008; Baucheron et al. 2012; Yamasaki et al. 2013). Every cefotaxime-evolved population accumulated mutations in *ramR*; however, they never reached fixation (fig. 1*B*; supplementary table S4, Supplementary Material online). These mutations were found both in the DNA-binding domain (RamR_10–49_), which interacts upstream of *ramA* to repress *ramA* transcription, and the substrate binding domain (RamR_50–193_) acting as a cytosolic multidrug sensor (Yamasaki et al. 2013). Finally, all five cefotaxime-evolved populations obtained mutations in STM14_RS01190 coding for a peptidoglycan glycosyltransferase. These mutations were fixed in three out of five cefotaxime-evolved populations (fig. 1*B*; supplementary table S4, Supplementary Material online). This gene shows at least 88% sequence similarity with the *ftsI* gene in *E. coli* which codes for the penicillin-binding protein 3, the target of β-lactam antibiotics such as cefotaxime (Sauvage et al. 2014; Sun et al. 2014; Adler et al. 2016). Moreover, BlastP revealed that the protein sequence is for 96% homologous to *E. coli* (Altschul et al. 1990; Madden 2003; Agarwala et al. 2018). Therefore, STM14_RS01190 will be designated *ftsI* from here on. All mutations in *ftsI* are located in the penicillin-binding domain (ftsI_237–587_) (Sauvage et al. 2014; Sun et al. 2014). Strikingly, controls C, D, and E also accumulated mutations in *ftsI*; in Pro311Leu in 47% and Gly481del in 0.7% of control C, in Ser85Gly in 88% of control B, and in Pro524Gln in 11% of control D. However, these mutations differ from the ones present in the cefotaxime-exposed lineages, except for Gly481del in control C. Although mutations Pro311Leu and Pro524Gln are also located in the penicillin-binding domain, these mutations are not necessarily associated with increased resistance of the controls (fig. 1*B*). Indeed, previous research also observed fixation of *ftsI* mutations in *E. coli* during experimental evolution in the absence of antibiotics and these mutations did not induce enhanced resistance against β-lactam antibiotics (Knöppel et al. 2017; Knöppel et al. 2018). Altogether, these data suggest that *ftsI* also has a role in growth medium adaptation (Knöppel et al. 2018).

From the remaining 22 out of 59 mutations in the cefotaxime-treated populations, only one mutation reached fixation (supplementary table S4, Supplementary Material online). This mutation was found in *ftsW* (99.52% in population CTX D), which is known to interact with *ftsI* in *E. coli* (Leclercq et al. 2017). The remaining mutations in the CTX-treated populations did not reach fixation in any population (highest allele frequency was only 47.66%), although parallelism was observed for a few of these mutations which might still suggest a role in adaptation. Indeed, populations CTX A (8%), CTX B (13%), CTX D (8%), and control E (11%) accumulated exactly the same single nucleotide polymorphism mutation in *bigA*. BigA functions as an adhesin, that has however not been associated with resistance before. In addition, pathway level parallelism was observed in populations CTX A, CTX C and CTX E, which accumulated mutations in three genes (*moaD*, *moaE*, and *moeB*, respectively) involved in the molybdopterin biosynthesis pathway. These genes, except *moaE*, were however also mutated in every control population (supplementary table S4, Supplementary Material online), suggesting that these mutations play a role in growth medium adaptation rather than resistance evolution. Control populations did not accumulate further mutations showing pathway parallelism that were not present in cefotaxime-treated populations. Overall, the data thus suggest that the mutations in *envZ*, *acrB*, *ramR*, and *ftsI* are the main drivers for cefotaxime resistance (supplementary table S4, Supplementary Material online). These findings will be further supported by the mutational dynamics over time, which will be described further on.

#### Cross-resistance is Omnipresent in Cefotaxime-Treated Populations

Before studying the effect of cefotaxime preadaptation on resistance evolution during treatment with a set of second antibiotics, we first determined the initial levels of cross-resistance and collateral sensitivity against these antibiotics. Hereto, we selected 12 antibiotics, spanning a wide range of common antibiotics and antibiotic classes (table 1). Cross-resistance occurred in 70% (42/60) of the cefotaxime-treated populations (fig. 1*C*). Moreover, cross-resistance levels against individual antibiotics did not deviate with more than one MIC dilution between parallel evolved populations, indicating high repeatability. As expected, cefotaxime-treated populations showed the highest levels of cross-resistance against the β-lactam antibiotics ampicillin and cefoxitin, except for population CTX A which did not show any cross-resistance against both antibiotics (fig. 1*C*; supplementary table S5, Supplementary Material online). Remarkably, we also observed increased resistance levels in 33.33% (20/60) of the control lineages (fig. 1*C*; supplementary table S5, Supplementary Material online). This increased resistance in the controls might be due to an elevated standing genetic variation compared with the ancestors as the controls accumulated 95 mutations occurring with frequency >10% compared with their ancestors (supplementary table S3, Supplementary Material online). In line with this, the hypermutator populations of controls B and D showed the highest incidence of resistance amongst all five control populations. These hypermutator phenotypes further increase the mutational variety and consequently enhance the possibility that a resistant mutant resides in the population (Martinez and Baquero 2000; Barrick and Lenski 2013). However, while hypermutators also occur in nature (Jolivet-Gougeon et al. 2011; Tanner and Kingsley 2018; Sheng et al. 2020), these phenotypes could potentially bias our results due to the limited number of parallel lineages. If these lineages were removed from the dataset, only 19.44% (7/36) of the controls showed increased resistance (supplementary table S5, Supplementary Material online). Regardless of hypermutators, elevated resistance was significantly more common in the cefotaxime-treated populations compared with the controls (*P* ≤ 0.0001, Fisher's Exact test). In addition, for the nine antibiotics against which an increased resistance was observed, the resistance levels were significantly higher for the cefotaxime-treated populations than for the controls with or without hypermutators (*P* < 0.0001 for both conditions; Wilcoxon rank-sum test).

**Table 1.** Overview of Antibiotics Used Throughout This Study.

Name	Abbreviation	Class	Target	Type	Solvent
**Cefotaxime**	CTX	β-Lactam	Cell wall synthesis	Bactericidal	Water
**Ampicillin**	AMP	β-Lactam	Cell wall synthesis	Bactericidal	50% ethanol
**Cefoxitin** **Ciprofloxacin**	CXT CIP	β-Lactam Fluoroquinolone	Cell wall synthesis DNA gyrase	Bactericidal Bactericidal	DMSO Water (HCl)
**Nalidixic acid**	NAL	Quinolone	DNA gyrase	Bactericidal	Water
**Nitrofurantoin**	NIT	Nitrofuran	Multiple mechanisms	Bactericidal	DMSO
**Kanamycin**	KAN	Aminoglycoside	Protein synthesis, 30S	Bactericidal	Water
**Tobramycin**	TOB	Aminoglycoside	Protein synthesis, 30S	Bactericidal	Water
**Tetracycline**	TET	Tetracycline	Protein synthesis, 30S	Bacteriostatic	70% ethanol
**Doxycycline**	DOX	Tetracycline	Protein synthesis, 30S	Bacteriostatic	Water
**Chloramphenicol**	CHL	Amphenicol	Protein synthesis, 50S	Bacteriostatic	95% ethanol
**Erythromycin**	ERY	Macrolide	Protein synthesis, 50S	Bacteriostatic	95% ethanol
**Trimethoprim**	TMP	Aminopyrimidine	Folic acid biosynthesis	Bacteriostatic	DMSO

In contrast to cross-resistance, collateral sensitivity only occurred in a limited number of cefotaxime-treated populations (13.33%–8/60, fig. 1*C*; supplementary table S5, Supplementary Material online), whereas no increased susceptibility was observed in the control populations (*P* < 0.0233, Fisher's Exact test—fig. 1*C*; supplementary table S5, Supplementary Material online). In addition, the impact of collateral sensitivity on resistance levels was relatively weak, resulting in maximum a 2-fold reduction in resistance. In line with literature, collateral sensitivity occurred predominately to the aminoglycosides kanamycin and tobramycin (Imamovic and Sommer 2013; Lázár et al. 2013; Barbosa et al. 2019), although, the opposite, that is collateral sensitivity of aminoglycoside resistant strains against other drug classes, including β-lactam antibiotics, has been reported much more commonly (Imamovic and Sommer 2013; Lázár et al. 2013; Oz et al. 2014; Barbosa et al. 2019). In addition, population CTX A also developed collateral sensitivity to the macrolide erythromycin (fig. 1*C*). As a result, the cefotaxime-treated populations showed a significantly lower resistance against these three antibiotics compared with the control populations (*P* = 0.0001, Wilcoxon rank-sum test—supplementary table S5, Supplementary Material online). The same result was obtained when hypermutator strains B and D were removed from the dataset (*P* = 0.0032, Wilcoxon rank-sum test).

#### Cefotaxime Preadaptation Impacts Subsequent Resistance Evolution to Second Antibiotics

Multiple studies already highlighted the potential of antibiotic cycling based on collateral sensitivity networks in order to tackle the antibiotic resistance crisis (Imamovic and Sommer 2013; Lázár et al. 2013; Kim et al. 2014; Yen and Papin 2017; Imamovic et al. 2018; Barbosa et al. 2019) and a few even observed that antibiotic cycling can impair resistance evolution in evolution experiments (Imamovic and Sommer 2013; Kim et al. 2014; Yen and Papin 2017; Yoshida et al. 2017; Barbosa et al. 2019). To date, the underlying mechanisms that interfere with adaptation during treatment with second antibiotics after pre-exposure to a first antibiotic are, however, poorly understood. Often this diminished resistance evolution against second antibiotics is attributed to higher initial susceptibility levels against the second antibiotic due to collateral sensitivity. However, adaptation could also be influenced in other ways by the genetic background evolved during drug cycling, that is by all mutations accumulated during exposure to the first antibiotic, and their potential interplay with the emergence and accumulation of mutations needed to adapt to the second antibiotic. In order to analyze the contribution of such additional effects, we aimed to level out the effect of differences in initial sensitivity to the second antibiotic and assess potential contributions of the genetic background beyond collateral sensitivity. We specifically focused on the first switch between two antibiotics and measured extinction over time. To eliminate potential differences in initial sensitivity, we exposed the cefotaxime preadapted and control populations to different second antibiotics, but normalized the applied concentration for each individual second antibiotic based on its MIC against each individual population (cefotaxime pretreated or control). We chose to normalize based on MIC as this is standard practice in studies that need to standardize antibiotic concentrations for other purposes (Baym et al. 2016; Mehta et al. 2018; Santos-Lopez et al. 2019). In order to gain the most general insight, we did not limit our analysis to the antibiotics where collateral sensitivity was observed. Specifically, each population was exposed to 1.5 times the MIC of five antibiotics (ampicillin, tetracycline, chloramphenicol, ciprofloxacin, kanamycin) that represent different antibiotic classes and experience different levels of cross-resistance or collateral sensitivity (fig. 1*A*–*C*–table 1). The adaptive potential to the second antibiotic was then assessed by measuring the optical density (OD_595_) of the population after 1–3 days (depending on the antibiotic's stability) and the population was considered adapted if its optical density reached the threshold value of OD_595, blank_ + 4 × standard deviation. Otherwise the population was considered extinct. Next to being most relevant for clinical practice, this focus on survival/extinction rates has the key advantage of permitting high parallel experiments to assess repeatability. In total, 50 repeats per population and per antibiotic were evaluated, resulting in 2,500 evolved populations, among which 1,250 controls and 1,250 cefotaxime pretreated populations. To also elucidate the effect of the hypermutator phenotype, the results were analyzed with and without taking the hypermutator lineages (controls B and D) into account.

Remarkably, cefotaxime preadaptation significantly impacted subsequent resistance evolution to second antibiotics even if the applied antibiotic concentrations were adjusted to compensate for initial differences in resistance, and thus effects of collateral sensitivity and cross-resistance (fig. 1*D*; supplementary table S6, Supplementary Material online). Disregarding hypermutator populations, exposure of cefotaxime pretreated populations to ampicillin or tetracycline resulted in a significantly higher degree of resistance evolution and survival rate compared with the controls, despite the cefotaxime pretreated populations experiencing higher ampicillin and tetracycline concentrations (fig. 1*D*). In contrast, cefotaxime pretreated populations were less able to adapt to ciprofloxacin and chloramphenicol (fig. 1*D*), even though the cefotaxime pretreated populations initially showed higher resistance toward these second antibiotics (fig. 1*C*). Although the applied ciprofloxacin and chloramphenicol concentrations were normalized to 1.5 times the MIC for every population, the decreased adaptation of the cefotaxime preadapted populations could be the result of over-normalization due to a potentially nonlinear relation between antibiotic concentration and resistance evolution. To eliminate possible effects of nonlinearity in antibiotic concentration, the ciprofloxacin concentration was reduced to 1 × MIC for cefotaxime pretreated populations only, but kept at 1.5 × MIC for the control populations. Even at this lower antibiotic concentration, cefotaxime pretreated populations still showed a reduced adaptation compared with the controls (1.5 × MIC), albeit not significantly (supplementary fig. S3*A*, Supplementary Material online). This observation further supports that effects of the evolved genetic background on mutations needed to adapt to the second antibiotic are the main contributor to reduced resistance evolution for these populations, rather than differences in initial inhibition by the normalized antibiotic concentrations. Additionally, the cefotaxime pretreated populations also adapted less well to the presence of kanamycin (fig. 1*D*), an antibiotic toward which the cefotaxime pretreated lineages showed collateral sensitivity (fig. 1*C*). The inclusion of the hypermutator lineages did not influence these findings, indicating that the hypermutators did not significantly alter the adaptation to secondary antibiotics (supplementary fig. S3*B*and S4 and table S6, Supplementary Material online).

As antibiotic resistance evolution is clearly impacted by prior adaptations even if the treatment concentration is normalized, our results indicate that the genetic background of cefotaxime preadapted populations influences the emergence and spread of mutations required to develop resistance against the second antibiotics, independently of effects of collateral sensitivity or cross-resistance. On the one hand, mutations present in the genetic background might change the emergence of novel resistance mutations, for example by influencing the mutation rate under stress-induced conditions (Hall 1998). On the other hand, mutations accumulated in the genetic background during exposure to the first antibiotic might interact with mutations emerged during treatment with the second antibiotic. Both additive and epistatic interactions might occur. The former means that the effect of the mutations against both antibiotics are additive (Knopp and Andersson 2018; Barbosa et al. 2019); the latter that the combined effect of the mutations deviates from what is expected based on their individual effects. Recent systematic evaluations of engineered combinations of resistance mutations support the potential for both additive (Knopp and Andersson 2018; Barbosa et al. 2019), as well as positive and negative epistatic interactions to occur (Ward et al. 2009; Barbosa et al. 2019; Porse et al. 2020; Liakopoulos et al. 2021). Moreover, drug cycling between gentamicin and carbenicillin was found to result in additive interactions and negative epistatic interactions between mutations in *pmrB* and *nalC/nalD* with regard to resistance to carbenicillin and gentamicin, respectively (Barbosa et al. 2019).

### Effect of Cefotaxime Gradient Strength on Resistance Evolution Against Second Antibiotics

#### Cefotaxime Resistance Evolves Under Different Gradient Strengths

The cefotaxime resistant strains in the previous section were obtained by exposing populations to a slowly increasing antibiotic concentration gradient until they reached *C*_max_ after approximately 66 days. This setup is representative of situations where bacteria are experiencing a temporal antibiotic gradient with concentrations increasing over time or, by extension, where bacteria are gradually migrating through a spatial antibiotic gradient. Such antibiotic gradients are often found in the human body due to poor antibiotic penetration into tissues or due to periodic antibiotic administration (Martinez and Baquero 2000; Zhang et al. 2011; Hermsen et al. 2012; Andersson and Hughes 2014; Oz et al. 2014; Moreno-Gamez et al. 2015; Baym et al. 2016; Hol et al. 2016; Jahn et al. 2017; Harmand et al. 2018). In addition, antibiotic gradients may occur in biofilms where antibiotics migrate slowly through the biofilm matrix (Tseng et al. 2013) or in soil communities where bacteria produce antibiotics to compete with species occupying the same niche (Romero et al. 2011; Hol et al. 2016). Previous research already showed that the strength of the applied antibiotic gradient influences the time required for adaptation as well as the obtained resistance levels (Zhang et al. 2011; Baym et al. 2016). Moreover, differences in gradient strengths have also been shown to impact subsequent collateral sensitivity or cross-resistance to other antibiotics (Oz et al. 2014; Jahn et al. 2017). Therefore, we aimed to evaluate the impact of the applied cefotaxime gradient on our previous findings. We expected that more evolutionary trajectories will be open for populations exposed to weak gradients, resulting in an increased mutational diversity and selection for resistance-conferring mutations with a relatively low fitness cost, as was previously observed by Lindsey et al. (2013) and Jahn et al. (2017). In contrast, strong gradient strengths might select for high-impact mutations in order to survive the treatment even if this imposes a larger fitness cost (Oz et al. 2014; Jahn et al. 2017), resulting in less mutational diversity (Lindsey et al. 2013). Consequently, populations exposed to weak gradients might have a higher potential for adapting to second antibiotics due to their increased genomic diversity (and mutational interactions) and the lower fitness cost of acquired resistance mutations.

**Fig. 2.**
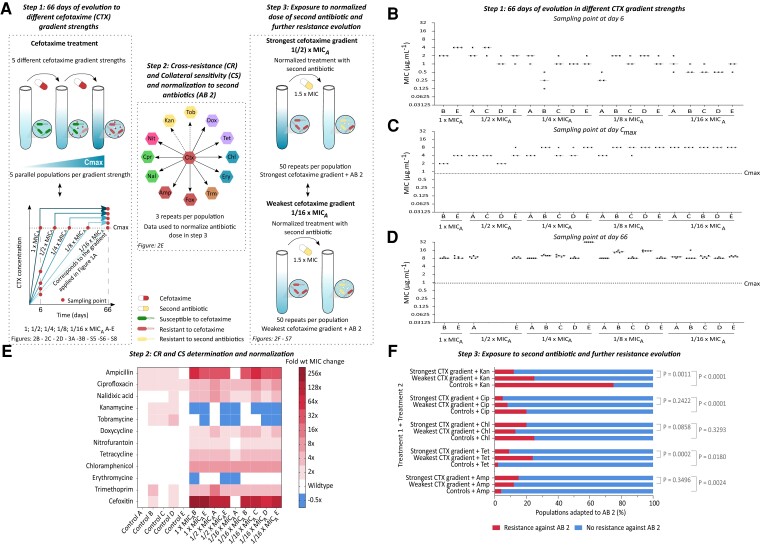
Gradient strength influences cefotaxime resistance levels and potential for adaptation to second antibiotics. (*A*) Experimental setup—Five ancestral clones were exposed to five linearly increasing cefotaxime gradients (step 1). Next, resistance levels against 12 additional antibiotics were determined using a MIC assay in step 2. These data were normalized to 1.5 × MIC for every population. Based on these normalized data from step 2, the populations previously exposed to the strongest or weakest cefotaxime gradient strength were evolved against five additional antibiotics (ampicillin, tetracycline, chloramphenicol, ciprofloxacin, and kanamycin) in step 3. (*B*) Cefotaxime resistance levels (µg/mL) at day 6 are higher for the populations exposed to the stronger cefotaxime gradient strengths, while (*C*) at day *C*_max_ resistance levels seem to increase with a decreasing gradient strength. (*D*) Between day *C*_max_ and day 66, cefotaxime resistance levels kept increasing, resulting in similar resistance levels across all populations, regardless of the applied gradient strength. In order to measure the MIC more accurately the resolution of the MIC assay at day 66 was increased to steps of 1 µg/mL between 8 and 16 µg/mL instead of using the standardized 2-fold dilution series. Horizontal lines indicate the median MIC of at least three independent biological repeats (*n* ≥ 3), each data point represents the MIC of one independent biological repeat. Dashed line indicates *C*_max_, the maximum concentration to which the populations were exposed (4 × MIC_A_ or 0.95 µg/mL). (*E*) Heatmap showing resistance levels against 12 antibiotics between populations evolved in the presence of the strongest and weakest cefotaxime gradient strengths and control populations. No differences in cross-resistance (*P* = 0.6462) or collateral sensitivity (*P* = 0.5626) were observed across different cefotaxime gradient strengths. Color scales indicate fold increase (cross-resistance) or decrease (collateral sensitivity) in antibiotic resistance relative to an untreated ancestral *S.* Typhimurium. *P*-values were derived from the Fischer's Exact test. (*F*) The gradient strength in which populations were pretreated with cefotaxime impacts the potential to adapt to subsequent antibiotics. Populations that were adapted to the weakest cefotaxime gradient strength showed more adaptation compared with the populations exposed to stronger cefotaxime gradients when exposed to kanamycin and tetracycline. In contrast, no significant differences in adaptation were found between cefotaxime gradient strengths when exposed to chloramphenicol, ciprofloxacin, and ampicillin. Shown are the percentages of populations able to adapt to the second antibiotic; cefotaxime (CTX), ampicillin (Amp), tetracycline (Tet), chloramphenicol (Chl), ciprofloxacin (Cip), and kanamycin (Kan). *P*-values are derived from a Fisher's Exact test. The data were analyzed after exclusion of the hypermutator populations. Inclusion of hypermutators removed the differences observed between the strongest and weakest cefotaxime gradients when kanamycin or tetracycline was applied as second antibiotic (supplementary fig. S7, Supplementary Material online). Additional source data underlying these figures can be consulted in supplementary tables S5, S6, S9, and S10, Supplementary Material online.

Thus, to further expand on how antibiotic gradients influence resistance evolution and to investigate whether subsequent adaptation to other antibiotics is also impacted, we first evolved five parallel *S*. Typhimurium lineages for 66 days in four additional, steeper, cefotaxime gradients. We started from the same five ancestral clones used to initiate the weakest gradient and the controls described before. In each concentration gradient the cefotaxime concentration was increased with steps equal to a constant fraction of the ancestral MIC (MIC_A_ = 0.24 µg/mL) (supplementary fig. S1 and table S1, Supplementary Material online), resulting in the following gradients: 1 × MIC_A_, 1/2 × MIC_A_, 1/4 × MIC_A_, 1/8 × MIC_A_ and 1/16 × MIC_A_ (fig. 2*A*). In total, the evolution experiment thus consisted of 30 populations, including the controls and the five cefotaxime-treated populations described above (CTX A–E which will be labeled 1/16 × MIC_A_ A–E from here on), in which each gradient strength is represented by five parallel lineages. Since bacteria can only colonize niches with a higher antibiotic concentration once they have acquired the necessary adaptations, we allowed the cells to adapt to the applied cefotaxime concentrations before increasing the concentration. This process was repeated until the populations reached the same maximum concentration (*C*_max_) of 4 × MIC_A_. Populations evolved under the stronger gradient strengths thus reached *C*_max_ faster than the populations subjected to the weaker gradient strengths (supplementary table S7, Supplementary Material online). To correct for these time differences, the lineages were afterwards maintained at *C*_max_ until the endpoint (day 66) where the last population (1/16 × MIC_A_ B) reached *C*_max_ (fig. 2*A*). The populations were checked for contaminations at every time point. As a consequence, population 1/2 × MIC_A_ B was completely removed from further analysis and populations 1/2 × MIC_A_ C and 1/2 × MIC_A_ D were removed from analysis at day 66, since they contained a *Bacillus* spp. contamination. The following sections focus on the impact of changing gradient strengths on resistance evolution against the initial applied antibiotic cefotaxime. Later we discuss the impact on resistance against second antibiotics.

#### Cefotaxime Gradient Strength Negatively Impacts Resistance Evolution

We started our analysis by comparing the survival rate of the populations subjected to different cefotaxime gradient strengths. Three out of five populations exposed to the strongest gradient (1 × MIC_A_) went extinct before reaching day *C*_max_ (i.e., the day that *C*_max_ [4 × MIC_A_] was reached). This day is different for every population, whereas all other lineages survived (supplementary table S7, Supplementary Material online). To investigate whether this high extinction rate of the strong gradient populations is a sampling artifact due to the small number of populations or whether the harsh initial conditions are responsible, 20 additional populations were evolved under the strongest increasing gradient (1 × MIC_A_). In total, 8 out of 25 lineages exposed to 1 × MIC_A_ went extinct (supplementary table S8, Supplementary Material online), resulting in a significantly lower survival rate (68% survival) compared with the survival rate of the populations subjected to the other gradients (*P* = 0.0065, Fisher's Exact Test). Indeed, all 19 populations evolved to the other gradient strengths survived (100% survival), most probably because they were initially subjected to sublethal cefotaxime concentrations, giving them the opportunity to acquire and select resistance-conferring mutations. In contrast, populations evolved under strong gradients were immediately exposed to the MIC giving them less opportunity to acquire the resistance-conferring mutations necessary for growth (Lindsey et al. 2013; Jahn et al. 2017).

Next, we compared the level of cefotaxime resistance, the fitness cost of resistance, and the mutational diversity between the different gradient strengths as these parameters are expected to influence adaptation to treatment with a second antibiotic (Barbosa et al. 2019). We performed this analysis for populations isolated at two different time points. A first time point is the day the lineage reached the maximum concentration (day *C*_max_). However, as the time required to reach this concentration strongly differs between the various gradient strengths (supplementary table S7, Supplementary Material online), we also analyzed all populations at the time point the weakest gradient reached these maximum concentrations (day 66). In addition, we determined the resistance levels and mutations at day 6 (the day at which the steepest gradient reached *C*_max_) in order to be able to delineate the mutational dynamics, which contributes to the identification of adaptive mutations (see further).

At day 6, the populations evolved under the stronger gradient strengths showed higher resistance levels compared with the populations exposed to the weaker gradient strengths (fig. 2*B*; supplementary table S9, Supplementary Material online). However, the overshoot in resistance, the ratio of the observed resistance level to the exposed cefotaxime concentration, was higher in populations evolved under weaker gradients than strong gradients (supplementary table S9, Supplementary Material online). In contrast, at day *C*_max_, the resistance levels of the populations evolved under a strong gradient strength (1 × MIC_A_ and 1/2 × MIC_A_) were remarkably lower (ranging from 2 to 4 µg/mL) than populations evolved under an intermediate or weak gradient strength (1/4 × MIC_A_, 1/8 × MIC_A_ and 1/16 × MIC_A_) (ranging between 4 and 8 µg/mL) (fig. 2*C*). The overshoot in resistance also remained higher for the weaker gradients (supplementary table S10, Supplementary Material online). These results suggest that, at the moment they reach the same cefotaxime concentration, weaker gradient strengths result in higher resistance levels. At the final time point, day 66, resistance levels further increased starting from the level observed at day *C*_max_ for almost every population (fig. 2*D*). The strongest increase in resistance was observed for the populations subjected to the strong gradient strengths 1 × MIC_A_ and 1/2 × MIC_A_, while populations evolved to 1/16 × MIC_A_ showed only a slight increase in resistance. Eventually, all lineages obtained similar resistance levels at day 66 regardless of the applied cefotaxime gradient (fig. 2*D*).

**Fig. 3.**
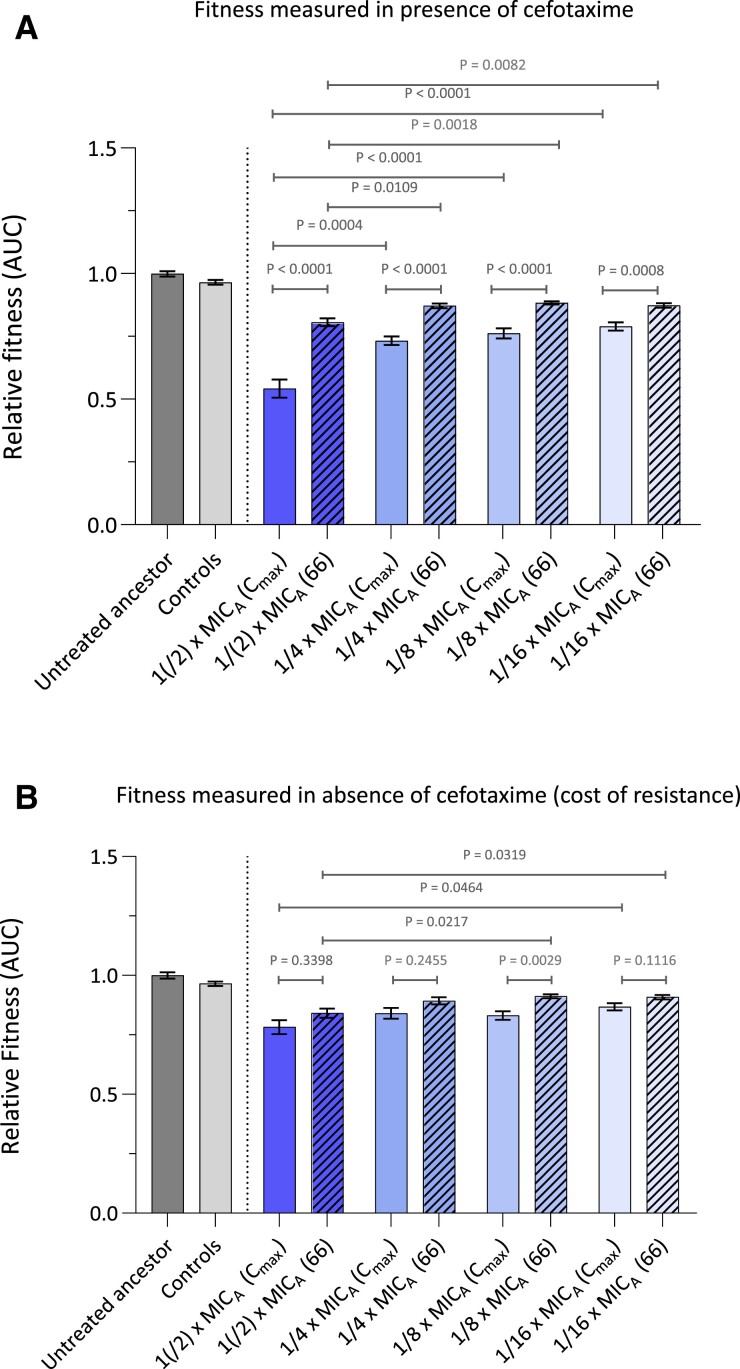
Fitness increases between day *C*_max_ and day 66 both in the presence and absence of cefotaxime, but remains below the level of the untreated ancestral strain. Fitness (*A*) in the presence and (*B*) in the absence of 4 × MIC_A_ (0.95 µg/mL) cefotaxime is measured as the area under the growth curve, and expressed relative to an untreated ancestral *S.* Typhimurium. Relative fitnesses at day *C*_max_ and day 66 are represented by open and filled bars, respectively. Color gradient indicates the applied cefotaxime gradient strength. Data from the strongest gradient strengths (1 × MIC_A_ and 1/2 × MIC_A_) were pooled together to obtain sufficient data points. *P*-values were derived from ANOVA or Welch ANOVA if standard deviations were significantly different (*P* < 0.5) and followed by Dunnett's (T3) (compare groups with a control group)—Tukey's (compare every group with every other group) or Sidak's (selected set of comparisons) multiple comparisons test. *P*-values related to comparisons with the untreated ancestral strain can be found in supplementary tables S11 and S12, Supplementary Material online. Error bars denote SEM, *n* = 3. Source data are provided in supplementary tables S11 and S12, Supplementary Material online.

Previous paragraphs indicated that the populations at day *C*_max_ already showed higher cefotaxime MIC levels compared with the applied cefotaxime concentration (4 × MIC_A_). Nevertheless, the cefotaxime resistance levels continued to increase between day *C*_max_ and day 66, while the administered cefotaxime concentration was held constant at 4 × MIC_A_ (fig. 2*C*–*D*). These observations suggest that there was still selection pressure for new or additional mutations after reaching *C*_max_. In order to provide additional support, the fitness at day *C*_max_ and day 66 was determined by growth curve analysis (area under the growth curve) in the presence of cefotaxime for each population evolved in each cefotaxime gradient strength. Data were pooled together per cefotaxime gradient strength. In order to obtain sufficient data points, the data from 1 × MIC_A_ and 1/2 × MIC_A_ were grouped together. The fitness levels at day *C*_max_ were found to be significantly lower than the untreated ancestral strain for all gradient strengths, but significantly improved between day *C*_max_ and day 66, suggesting that the populations indeed acquired additional mutations that increased their growth and MIC in the presence of cefotaxime (fig. 3*A*; supplementary table S11, Supplementary Material online). The fitness at day 66, however, remained below the level of the untreated ancestor and control evolved populations, indicating that further adaptation could be possible (fig. 3*A*; supplementary table S11, Supplementary Material online). Hereby the fitness levels of the strongest gradient strength (1(/2) × MIC_A_) was on average significantly lower than the intermediate (1/4 × MIC_A_ and 1/8 × MIC_A_) and weakest cefotaxime gradient strength (1/16 × MIC_A_) at both time points (fig. 3*A*; supplementary table S11, Supplementary Material online). Similar observations were found if we analyzed the fitness levels of the evolved populations separately (supplementary fig. S5*A*, Supplementary Material online).

#### Cefotaxime Gradient Strength is Positively Related to Fitness Cost of Resistance

A high fitness cost of resistance has previously been associated with an impaired adaptation to secondary treatment (Barbosa et al. 2019). Therefore, we here determined the fitness cost of cefotaxime resistance by measuring the growth curves in the absence of cefotaxime. As expected, the fitness levels at day *C*_max_ were found to be significantly lower as compared with the ancestral strain for all cefotaxime gradient strengths (pooled data analysis; fig. 3*B*; supplementary table S12, Supplementary Material online) and the majority of the cefotaxime-evolved populations separately (supplementary fig. S5*B*and table S12, Supplementary Material online). Populations exposed to the strongest cefotaxime gradient had on average a lower fitness than populations that were exposed to the weakest gradient strength, which supports the hypothesis that strong gradients initially select for costly high-impact mutations (1(/2) × MIC_A_ vs. 1/16 × MIC_A_ at day *C*_max_; *P* = 0.0464—Welch ANOVA). This observation is corroborated by previous work describing that populations evolved under strong selection pressures show lower fitness (expressed as growth rates) as compared with populations exposed to a mild selection pressures, indicating that strong selection pressures select for survival while mild pressures favor fitness (Lindsey et al. 2013; Oz et al. 2014; Jahn et al. 2017). The fitness levels were found to increase between day *C*_max_ and day 66 in almost every cefotaxime gradient strength (pooled data analysis fig. 3*B*; supplementary table S12, Supplementary Material online) and cefotaxime-evolved population (supplementary fig. S5*B*and table S12,, Supplementary Material online), albeit not significantly. This suggests that the populations obtained compensatory mutations that ameliorate bacterial fitness or replaced costly initial resistance mechanisms by mechanisms that impose a lower fitness cost (Andersson and Hughes 2010; Schulz zur Wiesch et al. 2010). However, the cost for resistance could not be fully compensated as fitness levels remained lower than that of the ancestral strain, an effect that was most outspoken for the stronger gradients (1(/2) × MIC_A_ vs. 1/16 × MIC_A_ at day 66; *P* = 0.0319—Welch ANOVA). Previous work suggests that this higher resistance cost in stronger gradients might negatively impact adaption to second antibiotics (Yoshida et al. 2017; Barbosa et al. 2019).

#### Cefotaxime Gradient Strength is Inversely Related to Diversity in Adaptive Mutations

We assessed the impact of the cefotaxime gradient strength on the mutational patterns associated with resistance acquisition to cefotaxime in order to link these patterns to the adaptive potential for resistance acquisition to the second antibiotics. In order to identify the adaptive mutations in the different gradient strengths, we evaluated the parallelism across the different lineages and mapped the mutational trajectories over time. Hereto, populations were whole genome sequenced at three time points. Next to day *C*_max_ and day 66, we also sequenced the populations at day 6, the day on which the populations subjected to the strongest gradient reached the maximum cefotaxime concentration. Populations exposed to 1/8 × MIC_A_ were however not sequenced at day 6 as we were mainly interested in the differences between the strongest (1(/2) × MIC_A_) and weakest gradients (1/16 × MIC_A_). In total, 17,275 mutations over all sequenced populations were found compared with the reference strain *S*. Typhimurium (for additional information see Materials and Methods and supplementary table WGS, Supplementary Material online). After filtering, (i.e., removing, synonymous mutations, mutations occurring in <0% of all sequenced populations, and mutations initially present in the ancestors) 650 mutations were retained, of which 548 in the cefotaxime-exposed lineages. Removal of hypermutator strains further reduced this number to 351, of which 310 in the cefotaxime-exposed lineages (supplementary table WGS, Supplementary Material online). On average, the total number of mutations per sequenced population compared with the ancestors continued to increase during the evolution experiment (supplementary table S13, Supplementary Material online), which further confirms that the populations further adapted to the imposed treatment conditions. However, in a minority of populations this number and thus also the heterogeneity in the population declined between day *C*_max_ and day 66.

The evolutionary dynamics showed that all populations exposed to cefotaxime acquired high frequency mutations (allele frequency above 50%) in *envZ–acrB–ramR–ftsI,* irrespective of the applied gradient strength. These mutations were also observed previously at the weak cefotaxime gradient, further supporting that these four genes contain adaptive mutations for cefotaxime resistance (supplementary tables S14–S18, Supplementary Material online). Moreover, after filtering and discarding hypermutators (1/2 × MIC_A_ A, 1/4 × MIC_A_ E, 1/8 × MIC_A_ B and 1/8 × MIC_A_ D), mutations in these four genes accounted on average for 57% of the total number of filtered mutations per sequenced time point (supplementary table S19, Supplementary Material online). In nearly every population, *envZ* and *acrB* mutations were already present in the cefotaxime-treated populations at day 6 (table 2; supplementary fig. S6*A*–*B*and table S14, Supplementary Material online) and they reached fixation in the majority of the populations at day *C*_max_ and day 66. Similar to the mutational analysis in the paragraphs above, the majority of the mutations were found in the cytoplasmic domain (43/79 mutations) (EnvZ_223–450_) and second transmembrane domain (22/79 mutations) (EnvZ_163–179_) of EnvZ. However, 13 out of 79 mutations were located in the periplasmatic region (EnvZ_35–162_), which acts as a osmolarity sensor and only one mutation was found in the first transmembrane domain (Tanaka et al. 1998; Inouye et al. 1999; Khorchid et al. 2005; Kishii et al. 2007). In AcrB, 47 out of 70 mutations were found in the periplasmic regions. The remaining mutations were found in the cytoplasmic (4/70 mutations) or transmembrane regions (19/70 mutations) (Baucheron et al. 2004; Yamaguchi et al. 2015). These observations, indicate that mutations in *envZ* and *acrB* are critical for the initial cefotaxime resistance evolution. Next, cefotaxime-treated populations acquired also mutations in *ramR*, which were mainly fixed in the strongest (1 × MIC_A_ and 1/2 × MIC_A_) and intermediate cefotaxime gradient strengths (1/4 × MIC_A_ and 1/8 × MIC_A_) after 66 days of evolution (table 2; supplementary fig. S6*C*and tables S14–S18, Supplementary Material online). In the weakest cefotaxime gradient strength (1/16 × MIC_A_) multiple clones were still competing (table 2; supplementary fig. S6*C*, Supplementary Material online). Except for one mutation (Lys5fs) these mutations were all located in the DNA-binding domain (54/88 mutations) or sensor domain (33/88 mutations) of RamR (Yamasaki et al. 2013). Mutations in *ftsI* were the last ones to accumulate, although this gene was predominately mutated in the weakest (1/16 × MIC_A_) and intermediate gradients (1/4 × MIC_A_ and 1/8 × MIC_A_) at day *C*_max_ (supplementary fig. S6*D*, Supplementary Material online). The presence of an *ftsI* mutation correlated positively with the resistance level at day *C*_max_, the causality of which was confirmed experimentally (see Addendum in supplementary Data, Supplementary Material online). Strikingly, at day 66, all populations accumulated mutations in the penicillin-binding domain of *ftsI* (Sauvage et al. 2014; Sun et al. 2014), even the populations that lacked a mutation in this gene at timepoint *C*_max_ (table 2; supplementary fig. S6*D*, Supplementary Material online). This suggests that *ftsI* mutations require longer exposure time and probably contribute to the enhanced resistance observed at day 66 compared with day *C*_max_.

**Table 2.** Frequency of Mutations Occurring in the Four-Key Driver Genes of Resistance at Three Different Time Points During the Evolution Experiment.

	1 × MIC_A_		1/2 × MIC_A_				1/4 × MIC_A_					1/8 × MIC_A_					1/16 × MIC_A_				
	B	E	A	C	D	E	A	B	C	D	E	A	B	C	D	E	A	B	C	D	E
*Day 6*																					
*envZ*	99.5	99.4	52.3	99.4	100.0	93.3	62.4	96.3	95.3	45.7	83.7						57.3	7.3	100.0	57.0	36.9
			49.0				25.0		2.6	4.9	13.5						13.3		4.2	36.4	20.1
							4.8										10.0			7.7	
							3.4										3.2				
																	2.2				
*acrB*	99.5	99.6	50.9	97.4	93.4	99.5	67.4	39.5	91.2	100.0	83.9							85.9			
			45.6				20.5	6.2			81.4										
											12.8										
											1.9										
*ramR*		45.0	82.6	96.6	51.5	97.1	3.7	85.6		36.5	53.0										
		41.3	13.6	1.8	29.2			4.6			22.7										
		11.7			3.6						12.3										
											6.2										
											4.6										
*ftsI*			3.9		6.3					47.8											
*Day C*_max_																					
*envZ*	99.5	99.4	84.8	99.2	97.9	99.3	94.5	99.6	99.5	100.0	87.7	99.5	99.8	100.0	99.7	100.0	99.3	99.5	100.0	99.4	100.0
			14.1								13.5										
*acrB*	99.5	99.6	81.4	92.8	50.4	97.9	94.4	74.3	99.2	100.0	83.9	99.7	99.5	98.1	62.5	59.3	99.4	99.0	90.3	99.6	99.8
			14.2	7.9	49.5		2.8	24.5			12.8				40.2	37.9			8.2		
											1.9										
*ramR*		45.0	82.6	82.8	51.5	97.1	93.1	100.0			52.9	99.7	20.4	81.8	44.5	99.2	38.2	24.0	92.5	73.6	34.7
		41.3	13.6	6.6	29.2		4.4				22.7		11.1	20.4	12.8		16.4	23.2	7.4	20.3	8.4
		11.7			3.6						12.3						6.4	10.5		5.2	7.4
											6.2						0.9	9.7			4.2
											4.6										2.1
*ftsI*			3.2	42.3	46.9		96.6		99.3	47.8	53.2	98.9	74.3	96.5	56.2	89.4	98.5	99.8	54.5	99.6	55.1
							4.2				13.4		21.9			39.7	8.5		35.0		43.9
											12.9								10.8		
																			4.8		
*Day 66*																					
*envZ*	99.9	100.0	96.6			99.7	94.4	100.0	96.8	100.0	87.6	99.7	99.9	100.0	99.9	100.0	99.2	99.5	100.0	99.4	100.0
			13.5				4.4				94.3				78.3						
			10.7												19.8						
			3.9																		
*acrB*	99.8	99.7	97.5			99.5	96.7	99.6	96.8	99.9	99.9	99.9	99.8	99.5	99.7	73.3	100.0	99.0	73.1	100.0	99.8
	58.1		3.5				3.4			83.3						21.7			20.8		
*ramR*		92.9	96.4			99.8	96.7	99.7	85.0		99.9	100.0	96.6	98.9	99.9	99.9	31.7	24.0	74.3	79.8	25.1
			3.7				3.1		2.5				1.9	2.6			31.6	23.2	25.2	20.6	14.6
									2.4								7.1	10.5			13.0
																		9.7			6.9
																					3.7
																					3.0
*ftsI*	99.6	94.8	96.8			99.9	93.1	99.8	97.7	99.9	100.0	99.6	96.2	97.7	99.9	91.9	99.9	99.8	55.4	99.7	64.5
		5.7	4.1				90.7		3.4				95.1		22.3	8.2			28.5	99.5	29.7
							4.2						2.5						11.9		
																			5.1		

NOTE.—The frequencies of the mutated variants are indicated in descending order per population and per gene; frequencies in one row do not necessarily represent the same variant.

In addition, several genes or metabolic pathways were repeatedly mutated, albeit at lower frequency and in a smaller number of populations. Indeed, mutations found in genes such as *ompR*, *tolA, rsxC,* and genes involved in molybdenum cofactor biosynthesis pathway (*moaA, moaD, moaE, moeA, moeB*) were mutated across parallel evolved lineages. Since these mutations occurred in multiple populations, they are likely also adaptive (supplementary tables S14–S18, Supplementary Material online). However, the majority of these variants displayed an allele frequency below 70% indicating that they are still competing with other mutations. Some of these mutations, such as those found in *ompR*, are directly related to β-lactam resistance whereas for the molybdenum cofactor biosynthesis, which was already identified in the analysis of the weak gradient, the direct link with antibiotic resistance is less clear (Kong et al. 2018). Moreover, the molybdenum cofactor biosynthesis genes were also mutated in the controls, suggesting that these mutations are important in growth medium adaptation. Next to adaptive mutations, some of the mutations occurring relatively frequently (allele frequency >10%) did not recurrently occur across parallel evolved populations (supplementary tables S14–S18, Supplementary Material online) indicating they were likely passenger mutations that got fixed by hitchhiking with adaptive mutations (Barrick and Lenski 2013; Tenaillon et al. 2016). Except in the hypermutator populations, very few of such passengers were observed.

Sequencing populations at multiple time points allows assigning mutations to individual clones and build mutational trajectories. This revealed that, rather than adapting to the imposed treatment conditions by sequential fixation through selective sweeps, the majority of the cefotaxime-treated populations showed clonal interference between different adaptive mutations. This competition between adaptive mutations can result in selection of less costly cefotaxime resistance mutations, which might explain the increase in fitness levels observed between day *C*_max_ and day 66. Moreover, the level of clonal interference between the four main resistance genes at day 66 but not at day *C*_max_ negatively correlated with the cefotaxime gradient strength, regardless of the presence of hypermutator 1/2 × MIC_A_ A (table 3 and supplementary fig. S6, Supplementary Material online, Fisher's Exact test, *P* = 0.0107). Mutations in these four genes have previously also been associated with enhanced resistance against several other antibiotics, such as ciprofloxacin (Baucheron et al. 2013; Lázár et al. 2014; Blair et al. 2015), ampicillin (Baucheron et al. 2004; Lázár et al. 2014; Adler et al. 2016; Adamowicz et al. 2020), tetracycline (Baucheron et al. 2004; Lázár et al. 2014), chloramphenicol (Baucheron et al. 2004; Toprak et al. 2012; Lázár et al. 2014), and kanamycin (Lázár et al. 2013; Hoeksema et al. 2019). In addition, on average the total number of mutations before filtering and the number of nonsynonymous mutations at day 66 (after removing hypermutator strain 1/2 × MIC_A_ A) were higher for populations exposed to weak cefotaxime gradient strengths compared with populations subjected to stronger cefotaxime gradients, although these results were not significant (table 3). Taken together, the higher levels of clonal interference and, to a lower extent, the difference in the number of mutations therefore suggest that weaker gradients might give rise to populations with a higher potential to adapt to subsequent antibiotics.

**Table 3.** At Day 66 Clonal Interference (CI) in the Four-Key Driver Genes of Antibiotic Resistance is More Prevalent in the Weakest Gradient Strengths compared with the Strongest Gradient Strengths.

	Day *C*_max_			Day 66		
	Cases of CI observed	Average number of total mutations	Average number of NS mutations	Cases of CI observed	Average number of total mutations	Average number of NS mutations
Strongest gradient: 1(/2) × MIC_A_	5/24	335.2 (274-355-316-278-404-384)	251.0 (215-273-244-235-269-270)	0/16^a^	228.3 (232-236-217)	188.3 (188-194-183)
Weakest gradient: 1/16 × MIC_A_	4/20	308.2 (314-307-253-236-356)	239.6 (235-244-278-192-249)	7/20	254.4 (232-307-253-236-244)	207.4 (199-244-203-194-197)
*P*-value	*P* > 0.9999^*^	*P* = 0.2155^**^	*P* = 0.5057^**^	*P* = 0.0107^*^	*P* = 0.2120^**^	*P* = 0.1801^**^

NOTE.—To obtain sufficient data, results from 1 × MIC_A_ and 1/2 × MIC_A_ were pooled. Also, the total number of mutations before filtering and nonsynonymous (NS) mutations, after removing ancestors, differed between both conditions.

a Result remains the same regardless of the presence of hypermutator 1/2 × MIC_A_.

* *P*-value obtained from a Fischer's Exact test.

** *P*-value obtained from an unpaired *t*-test.

#### Cefotaxime Gradient Strength Does not Impact Cross-resistance and Collateral Sensitivity Levels

Previous paragraphs showed that the stronger the cefotaxime gradient the larger the experienced fitness cost at both day *C*_max_ and day 66. However, it is only at day 66 that the populations subjected to the strong gradients displayed lower levels of clonal interference and mutational diversity than the populations subjected to the weak gradient strengths. We, therefore, selected the day 66 populations exposed to the strongest (1 × MIC_A_ and 1/2 × MIC_A_) or weakest (1/16 × MIC_A_) cefotaxime gradient strengths to further evaluate the impact of the cefotaxime gradient strength and hence the diversity in genetic background on the adaptive potential to the second antibiotics. We first studied the cross-resistance and collateral sensitivity levels against 12 different antibiotics (table 1–fig. 2*A*) via a MIC assay for these day 66 populations (fig. 2*E*). Again, cross-resistance levels against individual antibiotics did generally not differ more than one MIC dilution between parallel evolved populations, indicating high repeatability across parallel lineages. Moreover, cross-resistance incidence was found to be similar for both gradient strengths (*P* = 0.6462, Fisher's Exact test—supplementary table S5, Supplementary Material online) with 70% (42/60) of the combinations showing cross-resistance for the weak gradient strength and 75% of the combinations showing cross-resistance (27/36) for the strong gradient strengths (fig. 2*E*). Similarly, no differences were detected in the occurrence of collateral sensitivity (*P* = 0.5626, Fisher's Exact test—supplementary table S5, Supplementary Material online) with collateral sensitivity frequencies of 13.33% (8/60) and 19.44% (4 or 7/36) for the weak and strong gradients, respectively. Also no statistical differences in cross-resistance or collateral sensitivity were found when hypermutator population 1/2 × MIC_A_ A was included (supplementary table S5, Supplementary Material online). Moreover, the resistance levels of the antibiotics against which cross-resistance (*P* = 0.5446, Wilcoxon Rank-sum test) or collateral sensitivity (*P* = 0.3891, Wilcoxon rank-sum test) were observed, did not differ significantly between the weak and strong gradients, regardless of whether hypermutators were included or not (supplementary table S5, Supplementary Material online).

#### Cefotaxime Gradient Strength Impacts Resistance Evolution Against Second Antibiotics

Although we could not detect differences in cross-resistance and collateral sensitivity, we hypothesized that populations exposed to weaker gradients of the first antibiotic might adapt more easily to second antibiotics, since these populations show higher levels of clonal interference in the four resistance-associated genes and reduced cost of resistance against cefotaxime, which has been shown to positively impact adaptation to second antibiotics (Barbosa et al. 2019). Therefore, we challenged both populations that were exposed to the weakest (1/16 × MIC_A_) and strongest (1(/2) × MIC_A_) cefotaxime gradient strengths to five additional antibiotics (ciprofloxacin, chloramphenicol, tetracycline, ampicillin, kanamycin) in order to determine their adaptive potential against these antibiotics (fig. 2*A*). Again, the antibiotic concentrations were normalized based on the cross-resistance or collateral sensitivity level of the population in order to correct for differences in initial resistance levels and focus on the effects of the genetic background beyond collateral sensitivity (fig. 2*F*; supplementary table S6, Supplementary Material online). We analyzed 50 replicates per population and per applied antibiotic, resulting in a total of 3,500 evolved populations, including 1,250 controls, 1,000 strong gradients and 1,250 weak antibiotic gradients. As before the data were analyzed both with and without inclusion of the hypermutator populations.

Even though a correction factor was applied for initial differences in resistance, almost every population preadapted to cefotaxime showed an altered adaptation to the second antibiotics compared with the controls and the effect was clearly dependent on the cefotaxime gradient strength (fig. 2*F*; supplementary fig. S7 and table S6, Supplementary Material online). These data further support that the genetic background of the cefotaxime-treated populations strongly influences resistance evolution against the second antibiotic. Consistent with our hypothesis, populations exposed to the strongest cefotaxime gradients showed reduced adaptation toward tetracycline and kanamycin compared with the weakest gradient strength (fig. 2*F*), while no significant differences in adaptation between gradients were observed for the other antibiotics. These observations suggest that differences in clonal interference, mutational diversity, and cost of resistance between gradients of the first antibiotic drive subsequent adaptation to a subset of antibiotics. Inclusion of the hypermutator 1/2 × MIC_A_ A population neutralized the difference between strong and weak gradient strengths (supplementary fig. S7, Supplementary Material online), indicating that the strongly increased mutation rate overrules the effects of gradient strength.

## Discussion

Drug cycling is a promising approach to slow down resistance evolution and prolong the use of our currently available antibiotics. Previous research already showed the potential of drug cycling (Imamovic and Sommer 2013; Kim et al. 2014; Yen and Papin 2017; Yoshida et al. 2017; Barbosa et al. 2019), but it remained unclear to which extent these promising results were related directly to collateral sensitivity, or to additional effects of mutations accumulated in the genetic background during exposure to the first antibiotic on the emergence and spread of mutations during treatment with the second antibiotic. By normalizing the antibiotic concentration to eliminate effects of collateral sensitivity, we were able to demonstrate a clear contribution of the evolved genetic background beyond collateral sensitivity to the adaption against a second antibiotic, which either enhanced or reduced the adaptive potential depending on the specific drug combination. Mutations present in the genetic background might change the emergence of novel resistance mutations by altering the mutation rate (Hall 1998). Alternatively, mutations acquired during treatment with the first antibiotic might engage in additive or epistatic interactions with mutations emerged during treatment with the second antibiotic (Ward et al. 2009; Knopp and Andersson 2018; Barbosa et al. 2019; Porse et al. 2020; Liakopoulos et al. 2021). We further demonstrated that the applied antibiotic exposure pattern can have a strong impact on the level of clonal interference in key driver genes of resistance, mutational diversity, and the fitness cost of resistance, with stronger antibiotic gradients associated with lower levels of clonal interference and higher costs. These parameters might explain the lower adaptive potential to certain second antibiotics we observed in populations that were preadapted to stronger gradients of the first antibiotic.

The previous evolution experiments in which drug cycling was evaluated, provide further support for the importance of effects of the genetic background that go beyond collateral sensitivity, although these studies were not focused on elucidating such effects. Barbosa et al. (2019) selected 14 drug pairs showing collateral sensitivity and studied extinction rates in linearly increasing antibiotic concentrations of the second antibiotic after adaption to the first antibiotic. The study did however not include a direct comparison with control lineages that were not adapted to the first antibiotic, and the doses of the second antibiotic were only partially normalized based on the effect size of collateral sensitivity, that is the starting concentration of the gradient was normalized, but not the end concentration. These differences in set up aside, the results are still consistent with ours in the sense that clear differences in extinction rates were observed between the drug pairs (including drug pairs with the same second antibiotic) and these differences in extinction rates were not related to the effect size of collateral sensitivity. Directly consistent with our results of reduced resistance evolution against kanamycin in cefotaxime pretreated populations, populations initially exposed to β-lactam antibiotics were found to experience strong difficulties adapting to aminoglycosides. Other evolution studies focused on the effect of drug cycling on the obtained resistance levels, rather than on extinction rates. Yen and Papin (2017) cycled different drug pairs at long term intervals, whereas Kim et al. (2014) and Yoshida et al. (2017) performed short term cycling in order to evaluate the obtained resistance levels in time and compare these to mono-treatments. The first two studies applied the steepest antibiotic gradient allowing survival by serially transferring the populations in series of antibiotic concentrations, while the third study used a morbidostat to adjust the antibiotic concentration to ensure maintained exponential growth. These antibiotic-dosing regimens thus normalize the initial doses based on the effect size of collateral sensitivity, but normalization is only partial because the doses are adjusted in time based on the acquired adaptations, which are stochastic in nature. These studies nevertheless also provide support for our hypothesis because drug cycling was found to strongly affect both the resistance levels obtained as well as the evolved mutations, also for drug combinations where no collateral sensitivity or cross-resistance was present. Yen and Papin (2017) for example found that pre-exposure to piperacillin prior to tobramycin resulted in reduced tobramycin resistance evolution, despite the absence of collateral sensitivity between piperacillin and tobramycin. This effect was largely due to chromosomal deletions acquired during piperacillin pretreatment. Correspondingly, Kim et al. (2014) found that cycling between ciprofloxacin and neomycin resulted in a reduced ciprofloxacin resistance evolution compared with a treatment with only ciprofloxacin, although collateral sensitivity is absent between this antibiotic pair. Finally, Yoshida et al. (2017) showed that cycling with rifampicin reduced resistance evolution against polymyxin, despite rifampicin resistance showing cross-resistance toward polymyxin (Yoshida et al. 2017).

The lower adaptation potential to tetracycline and kanamycin we observed in populations preadapted to strong compared with weak cefotaxime gradients might at least be partly explained by the higher cost of cefotaxime resistance in populations exposed to strong gradients. Cost of resistance against a first antibiotic was indeed found before to be negatively associated with adaption to second antibiotics (Barbosa et al. 2019). The higher cost of resistance itself is consistent with previous work describing that populations evolved under strong selection pressures show lower fitness as compared with populations exposed to mild selection pressures, indicating that strong selection pressures select for survival while mild pressures favor fitness (Lindsey et al. 2013; Oz et al. 2014; Jahn et al. 2017). In addition, we hypothesize that the lower levels of clonal interference and mutational diversity observed in populations exposed to strong cefotaxime gradients also reduce the adaptation to second antibiotics. This reduced mutational diversity might be explained by selection of rare high-impact mutations in strong gradients, as compared with the more diverse evolutionary trajectories that could be explored in weak gradients (Lindsey et al. 2013).

Comparison of resistance and cross-resistance levels between populations exposed to weak and strong cefotaxime gradients as intermediate steps in our approach also yielded a number of insights that shed light on previous, seemingly contradictory outcomes in the literature. Specifically, Oz et al. (2014) indicated that strong antibiotic gradients result in higher resistance levels in comparison to weak gradients, whereas Jahn et al. (2017) showed that selection under strong or weak gradient strengths results in similar phenotypes. The former study, however, phenotyped the populations at a fixed time point but at different treatment concentrations (similar to day 6 in our setup), whereas the latter analyzed the populations at a fixed treatment concentration (similar to the analysis of day *C*_max_ in our setup). By analyzing multiple time points, our study showed that the effect of the antibiotic gradient is dependent on the choice of the time point to analyze. Indeed, weak gradients were associated with lower resistance levels than strong gradients at the early fixed time point (day 6), but strongly increased in resistance afterwards, resulting in higher levels of resistance in weak gradients at the time the maximum concentration *C*_max_ was reached. Further prolonging the antibiotic treatment in all populations till day 66, however, increased resistance in the strong gradients and leveled out differences between the gradients. Furthermore, Oz et al. (2014) showed that populations evolved under strong selection pressure acquired higher levels of cross-resistance, whereas Jahn et al. (2017) found no impact of gradient strength on cross-resistance or collateral sensitivity development. Again this might be explained by differences in time points analyzed, which were the same as outlined for the resistance levels above. Similar to the latter study, we analyzed cross-resistance at a later stage (day 66) in the antibiotic gradient and obtained a similar outcome, that is no effect of gradient strength on cross-resistance or collateral sensitivity. When translating to clinical practice, overall our results support the current clinical guidelines indicating that antimicrobial therapies should avoid weak antibiotic gradients in order to reduce the chance of adaptation and prevent high levels of resistance. In addition, prolonged antibiotic exposure should be avoided as we showed that this gives rise to additional resistance evolution and better-adapted populations (Paterson et al. 2016; Iosifidis et al. 2017).

In conclusion, our results highlight that not only collateral sensitivity and cross-resistance networks, but also additional effects of resistance mutations on the adaptive potential toward other antibiotics should be taken into consideration when designing drug cycling strategies, as well as their dependencies on the antibiotic exposure pattern.

## Materials and Methods

### Bacterial Strains, Media, and Growth Conditions

Ancestral clones used to initiate the evolution experiment were obtained from *Salmonella enterica*, subsp. *enterica* serovar Typhimurium ATCC14028 (Fields et al. 1986). Strains were grown overnight (ON) in lysogeny broth (LB) or colony factor antigen (CFA) (10 g/L casamino acids, 1.5 g/L yeast extract, 50 g/L MgSO_4_, and 5 g/L MnCl_2_ at pH 7.4) at 37°C with aeriation at 200 rpm or on solid CFA agar plates (15 g/L bacteriological agar). The antibiotics used in this study (table 1) were stored in aliquots at –20°C.

### Dose–response Curve for the Ancestor Strain (MIC_A_)

An ON culture of *S.* Typhimurium was normalized to an optical density at 595 nm (OD_595_) of 2.5 (Genesys 10S UV-VIS spectrophotometer, Thermo Fisher Scientific), which corresponds to a cell density of approximately 2.10^9^ cfu/mL. Subsequently, the normalized culture was diluted 1:200 in 5 mL CFA supplemented with varying cefotaxime concentrations (0.05; 0.10; 0.14; 0.19; 0.24; 0.48; 0.95; and 2.39 µg/mL). After 24 h incubation at 37°C and 200 rpm, the colony forming units (CFUs) were determined by serial dilution and plating. The MIC_A_ was defined as the lowest cefotaxime concentration that prevented growth.

### Experimental Evolution Under Different Cefotaxime Gradient Strengths

Five random *S*. Typhimurium clones (named “A”–“E”) were each evolved to five different cefotaxime gradient strengths in which the concentration was let to increase with a different fraction of the MIC_A_ (1 × MIC_A_, 1/2 × MIC_A_, 1/4 × MIC_A_, 1/8 × MIC_A_ and 1/16 × MIC_A_). Hereto, five ON cultures, originating from the five clones (“A”–“E”), were normalized to an OD_595_ of 2.5 and diluted 1:100 in test tubes containing 5 mL CFA. Each normalized cell culture was used to inoculate six different test tubes: one control without cefotaxime and five tubes with increasing concentrations of cefotaxime (see above), resulting in a total of 30 populations. These populations were incubated for 24 h at 37°C and 200 rpm.

After 24 h of incubation, a first fraction of each lineage was stored at −80°C in 25% glycerol. A second fraction was used to measure the OD_595_ of the populations. Populations for which the OD_595_ of the cefotaxime-exposed population was equal to or greater than 1/2 × OD_595_ of the antibiotic-free populations were diluted 1:100 in 5 mL CFA and the cefotaxime concentration was raised with one step in accordance to the applied concentration gradient (1 × MIC_A_, 1/2 × MIC_A_, 1/4 × MIC_A_, 1/8 × MIC_A_ and 1/16 × MIC_A_). If the criterion was not met, half of the population (2.5 mL) was washed twice in 1 × PBS (8.8 g/L NaCl, 1.24 g/L K_2_HPO_4_ and 0.39 g/L KH_2_PO_4_) and resuspended in 5 mL CFA. Subsequently, the same amount of cefotaxime as on the previous day was added. This resulted in a 2-fold dilution of the cells and gave them additional time to adapt to the imposed treatment conditions.

This process was repeated every day until the populations reached the maximum cefotaxime concentration (*C*_max_) of 4 × MIC_A_. After reaching *C*_max_, the populations were daily diluted 1:100 to fresh medium, while keeping the cefotaxime concentration constant at *C*_max_. All populations were evolved for a total of 66 days, the number of days at which the last population exposed to the weakest gradient strength reached *C*_max_.

All evolved populations were controlled regularly for contaminations during the evolution experiment as well as at the three analysis points (day 6, day *C*_max_ and day 66), by checking their colony morphology on CFA. Subsequently, doubtful colonies were characterized by Sanger sequencing the 16S rRNA (Eurofins Genomics—supplementary table S20, Supplementary Material online).

### Selecting Single Colonies From Evolved Populations

Populations evolved to cefotaxime were streaked on CFA agar supplemented with 0.95 µg/mL (4 × MIC_A_) cefotaxime after reaching *C*_max_. Subsequently, 10 single colonies were randomly selected and grown ON in 96-well microtiter plates in CFA supplemented with 0.95 µg/mL (4 × MIC_A_) cefotaxime and frozen at −80°C in 25% glycerol. Prior to MIC tests these frozen cultures were streaked on CFA agar supplemented with 0.95 µg/mL cefotaxime.

### MIC Assays

MIC assays were performed based on a standard linear broth microdilution protocol with minor modifications (Wiegand et al. 2008). Briefly, 100 µL of frozen population samples or ON cultures (*ftsI* mutants) were diluted in CFA to achieve a final inoculation density of ±5.10^5^ CFU/mL. Subsequently, the samples were inoculated in a 96-well microtiter plate filled with CFA and a 2-fold antibiotic dilution series. The 96-well plates were closed with a breathable sealing membrane (Greiner Bio-One) to avoid excessive evaporation during static incubation at 37°C. After 20–24 h incubation, the OD_595_ was measured using the Multimode synergyTM multireader MX (BioTek). The MIC was defined as the lowest antibiotic concentration at which the OD_595, population_ reached the threshold of: OD_595, population_ > OD_595, blank_ + 2×standard deviation, _blank_.

### *ftsI* Mutant Construction

Evolved populations at day *C*_max_ containing an *ftsI* mutation were reverted to allele of an ancestral *S*. Typhimurium strain via homologous recombination. First, random colonies were selected from populations 1/4 × MIC_A_ A–D, 1/8 × MIC_A_ A, and 1/16 × MIC_A_ B–D–E and the presence of a specific *ftsI* mutation was confirmed by Sanger Sequencing. In a second step, a kanamycin resistance (Kan^R^)-cassette was inserted at the 5′ site of the neighboring *mraZ* gene in the ancestral *S.* Typhimurium (Datsenko and Wanner 2000). Finally, the Kan^R^-cassette was transferred via phage P22 transduction to the selected *ftsI* containing colonies. The P22 head packages DNA fragments up to ± 44 kbp (Casjens and Weigele 2005), resulting in the transfer of the Kan^R^-cassette together with surrounding DNA. Selective plating on kanamycin allowed to select for transformants containing the Kan^R^-cassette. Next, 10 colonies per acceptor strain were randomly selected and screened for the presence of the wildtype *ftsI* allele. By performing MIC assays, we confirmed that the Kan^R^-cassette did not impact cefotaxime resistance (supplementary table S21, Supplementary Material online). The template and primers used for the construction of these mutants are listed in supplementary table S20, Supplementary Material online.

### Preparing Genomic DNA for Whole Genome Sequencing

In total, 68 populations were submitted to whole genome sequencing (WGS; 5 ancestors, 5 controls at day 6 and day 66, 14 populations at day 6, 21 populations at day *C*_max_, and 18 populations at day 66—supplementary table S13, Supplementary Material online). Genomic DNA (gDNA) was extracted from frozen population samples by thawing 100 µL of the frozen sample and adding 4.9 mL CFA supplemented with the appropriate cefotaxime concentration (supplementary tables S9, S10, Supplementary Material online). After 24 h incubation at 37°C and 200 rpm, gDNA was extracted using the Qiagen Blood & Tissue DNeasy kit (Qiagen). 100 µL nuclease free water was used to elute the gDNA from the binding column. The DNA concentration and quality of the gDNA was analysed using the Nanodrop® ND-1000 Spectrophotometer (Thermo Fischer Scientific). Subsequently, the samples were sent packaged in dry ice to Genewiz incorporation for Illumina Sequencing with a coverage of 800x.

### Further Analysis of WGS

Prior to aligning the sequencing reads to the reference genome; read quality was assessed using FASTQC, adapter sequences were removed using TrimGalore and the base quality was recalibrated using Dindel. Next, the sequencing reads were aligned to the *Salmonella enterica* subsp. *enterica* serovar Typhimurium str. 14028S reference genome (Accession number NC_016856; Version NC_016856.1; GI: 378448274). Variant calling was done by LoFreq-V2, which allowed the detection of low-frequency variants. Finally, SnpEff was used to predict the impact of a mutation on the protein sequence. Mutations found in the evolved populations compared with the reference strain are listed in supplementary table WGS, Supplementary Material online. Since the five ancestor clones “A”–“E”, used to initiate the evolution experiment, also acquired mutations compared with the reference strain, all cefotaxime-evolved populations and controls were analysed with reference to their respective ancestors, for example the genome of population 1 × MIC_A_ B was compared with the genome of ancestor B. After removing ancestral mutations, also synonymous mutations and mutations that are present in <10% of all sequenced populations were removed from further analysis.

### Adaptation to a Second Antibiotic

ON cultures were normalized to an OD_595_ of 2.5 in 1 × PBS and subsequently diluted in CFA, supplemented with the appropriate antibiotic concentration, to obtain a final inoculation density of 5.10^5^ CFU/mL. This solution was inoculated into 96-well plates and sealed with a breathable sealing membrane. Populations were subjected to an antibiotic concentration of 1.5 times the MIC of the applied antibiotics (ampicillin, tetracycline, chloramphenicol, ciprofloxacin, kanamycin), unless stated otherwise. The 96-well microtiter plates were incubated at a temperature of 37°C, while continuously shaken at 200 rpm for one to maximum three days. The duration was set to 60% of the reported antibiotic stability at 37°C (supplementary table S22, Supplementary Material online). After incubation, the OD_595_ was measured using the SynergyTM multimode reader MX. Populations were considered to have adapted, if the OD_595_ reached the cut-off value of OD_595, blank_ + 4 × standard deviation.

### Growth Analyses and Relative Fitness

Bacterial growth curves of day *C*_max_ and day 66 populations were determined in either the presence or absence of cefotaxime. Hereto, the OD_595_ of ON cultures was measured and normalized to an OD_595_ of 2.5 and subsequently diluted 1:1000 in CFA presence or absence of cefotaxime to obtain an inoculation density of 5.10^5^ cfu/mL. 300 µL of each sample was incubated in Honeycomb Microplates (Oy Growth Curves Ab Ltd) for 24 h at 37°C, continuously shaking at 200 rpm. The OD_595_ was measured every 15 min by using the Bioscreen C device (Oy Growth Curves Ab Ltd). In total, three biological repeats were performed, each consisting of five technical repeats. The relative fitness was then calculated as the ratio of the area under the curve (integral of the growth curve) between a cefotaxime-evolved population or a control population and an ancestral *S.* Typhimurium. The integral of the growth curve incorporates three growth parameters namely, the lag phase, the exponential growth rate and the carrying capacity (Dunai et al. 2019; Ram et al. 2019; Spohn et al. 2019). Prior to calculating the area under the growth curve, blanks containing only growth medium, were subtracted from the raw OD_595_ data. Afterwards, the data were scaled to zero (at time = 0) by taking the natural logarithm of OD_595_/OD_595, t = 0_. Hereto, negative values were removed by subtracting the lowest OD-value from the entire dataset.

### Criteria for Detecting Clonal Interference in Sequenced Populations

Only mutations present in the four main genes of interest (*ftsI, acrB, envZ* and *ramR*) with a frequency above 10% were taken into account to detect differences in levels of clonal interference between the strongest and weakest gradient strengths. Clonal interference was defined if multiple nonfixed mutations are present simultaneously in a gene.

### Statistics

All data were obtained from at least three independent biological repeats (*n*). Data from the MIC assay (fig. 1*B*) were analysed using the nonparametric Kruskal–Wallis test followed by the post hoc Dunn's multiple comparisons test. The Wilcoxon rank-sum test was used to detect differences in cross-resistance/collateral sensitivity levels between two groups (CTX pretreated vs. controls or strong CTX gradients vs. weak CTX gradients). The Fisher's exact test was used to detect differences between proportions of two categorical variables, specifically differences (i) in the proportion of cross-resistance/collateral sensitivity between CTX pretreated and control groups (fig. 1*C*) or between strong CTX gradients and weak CTX gradients (fig. 2*E*) and (ii) differences in adaptation toward second antibiotics (fig. 1*D*, fig. 2*F*, supplementary figs. S3, S4, S7, Supplementary Material online). Statistical differences in relative fitness (fig. 3 and supplementary fig. S5, Supplementary Material online) or the minimum inhibitory concentration of the ancestor (MIC_A_) (supplementary fig. S1, Supplementary Material online) were determined via the following approach: Normality of the parametric data was first tested using the D’Agostino-Pearson Omnibus test if *n* > 6, when *n* < 6 normal distribution was assumed. If data were normally distributed, One-Way ANOVA or Welch ANOVA if SD were significantly different (*P* < 0.5) was performed to compare multiple groups. Dunnett's T3 (performed after Welch ANOVA), Sidak's (Selected pairwise comparisons), Tukey's (compare every group with every other group), or Dunnett's (compare every group to a control group) multiple comparison tests were used as post hoc test to search for differences between the different groups. The unpaired Student's *t*-test with Welch's correction if SD was significantly different (*P* < 0.05) was applied to determine differences between two populations (table 3).

## Supplementary Material

Supplementary data are available at *Molecular Biology and Evolution* online.

## Supplementary Material

msac257_Supplementary_Data

## Data Availability

The data underlying this article are available in the article and in its online Supplementary material. Raw sequencing reads are available via the SRA repository of NCBI (www.ncbi.nlm.nih.gov/sra), Bioproject number PRJNA894947, samples SAMN31476771–SAMN31476838.
