# Supplementary material for: Antibiotic Cycling Affects Resistance Evolution Independently of Collateral Sensitivity
Source: Mol Biol Evol. 2022 Dec 8;39(12):msac257. doi: 10.1093/molbev/msac257 (PMC9778841; doi:10.1093/molbev/msac257)
Supplement: msac257_Supplementary_Data [file msac257_supplementary_data.zip › Addendum and Supp Figures.pdf]

Antibiotic cycling affects resistance evolution independently of collateral sensitivity

Addendum and Supplementary Figures

Author information:

Pauline Brepoels[(1)*]; Kenny Appermans[(1)*]; Camilo Andres Pérez[(2)]; Bram Lories[(1)]; Kathleen Marchal[(2)]; Hans Steenackers[(1)]

[*] These authors contributed equally

Affiliations:

(1) Department of Microbial and Molecular Systems, Centre of Microbial and Plant Genetics (CMPG), KU Leuven, Leuven, Belgium

(2) Department of Information Technology and the Department of Plant Biotechnology, Biochemistry and Bioinformatics, Ghent University, Ghent, Belgium

Corresponding author:

1. Hans Steenackers (hans.steenackers@kuleuven.be)

Addendum – *FtsI* mutations are the driving force for cefotaxime resistance

Whole genome sequencing of cefotaxime treated populations revealed that four genes (*envZ – acrB – ftsI – ramR*) were repeatedly mutated in almost every population, indicating that these genes were the main drivers for cefotaxime resistance (Table 2). Populations evolved in presence of a stronger (1(/2) x MIC_A) cefotaxime gradient were rarely mutated in the target of cefotaxime, *ftsI* (Sun et al. 2014), at day C_{max}, while populations evolved under intermediate (1/4 x MIC_A – 1/8 x MIC_A) and weak (1/16 x MIC_A) cefotaxime gradients accumulated more frequently an *ftsI* mutation (Table 2 -Supplementary Figure S6D). Moreover, populations without a mutation in *ftsI* showed a lower cefotaxime resistance level compared to populations that accumulated an *ftsI* mutation, suggesting that these mutations contribute to the pattern of increasing cefotaxime resistance with a decreasing gradient strength (Table 2 -Figure 2C - Supplementary Figure S6D). In contrast with this observation, population 1/4 x MIC_A B showed high resistance towards cefotaxime despite the lack of an *ftsI* mutation. However, this population was the only population with a fixed mutation in *cpxA*, encoding the sensor histidine kinase of the two-component system CpxR/CpxA (Supplementary Data Table S16). Mutations in *cpxA* have previously been linked to antibiotic resistance and could possibly explain the high resistance of this mutant (Sun et al. 2009; Jahn et al. 2017). Strikingly, at day 66, all populations accumulated mutations in *ftsI*, even the populations that previously lacked a mutation in *ftsI* (Table 2 – Supplementary Figure S6D). Moreover, these *ftsI* mutations are almost completely fixed in every population with the exception of populations 1/16 x MIC_A C and 1/16 x MIC_A E. Therefore, we validated the impact of *ftsI* mutations on cefotaxime resistance. *ftsI* mutations present in populations 1/4 x MIC_A A-D, 1/8 x MIC_A A and 1/16 x MIC_A B-D-E at day C_{max} were reverted to the wildtype allele of an ancestral *S*. Typhimurium. Afterwards, their resistance levels were measured via a MIC assay. The cefotaxime resistance levels of the evolved strains, which were reverted to the wildtype allele, decreased to the level of populations that did not accumulate a mutation in *ftsI* (Supplementary Figure S8). This observation combined with the fixation of the *ftsI* mutations at day 66 and the levelling out of the resistance levels further strengthens the hypothesis that *ftsI* mutations are responsible for the difference in resistance levels between different gradient strength populations at day C_{max}.

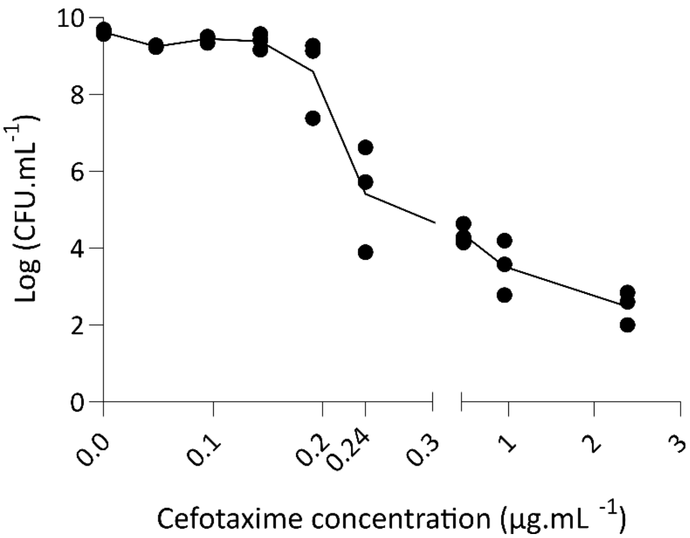

Supplementary Figure S1: Dose response curve of growth inhibition by cefotaxime for the ancestral *S.* Typhimurium ATCC 14028 strain. The minimum inhibitory concentration of the ancestor (MIC_A) was determined at 0.24 µg.mL^{-1}. Output of the statistical analysis as well as the source data can be found in Supplementary Data Table S1 and Supplementary Data Table S1 - Source Data, respectively. Each dot represents one independent biological repeat (n = 3). P-values were derived from one-way ANOVA followed by Dunnett's multiple comparisons test.

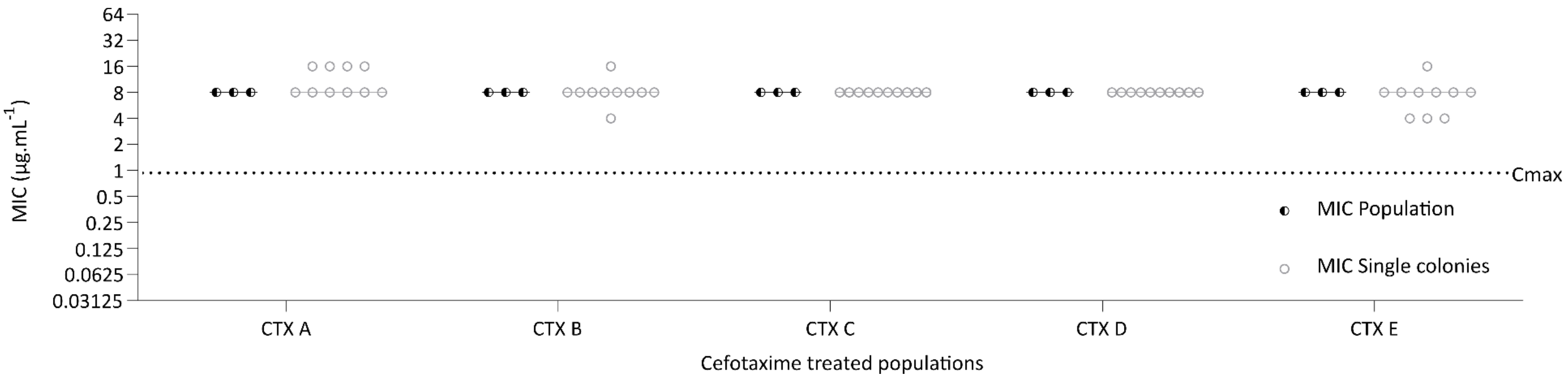

Supplementary Figure S2: Cefotaxime resistance levels at day C_{max} for cefotaxime exposed populations are similar between populations and ten randomly selected colonies from these populations. For the populations, each dot represents the MIC of one independent biological repeat. In case of the single colonies, each dot represents the median of at least three independent biological repeats per colony. Dashed line indicates C_{max}, the maximum concentration the populations were exposed (4 x MIC_A or 0.95 µg.mL^{-1}). Data of independent biological repeats per colony can be found in Supplementary Data Table S2.

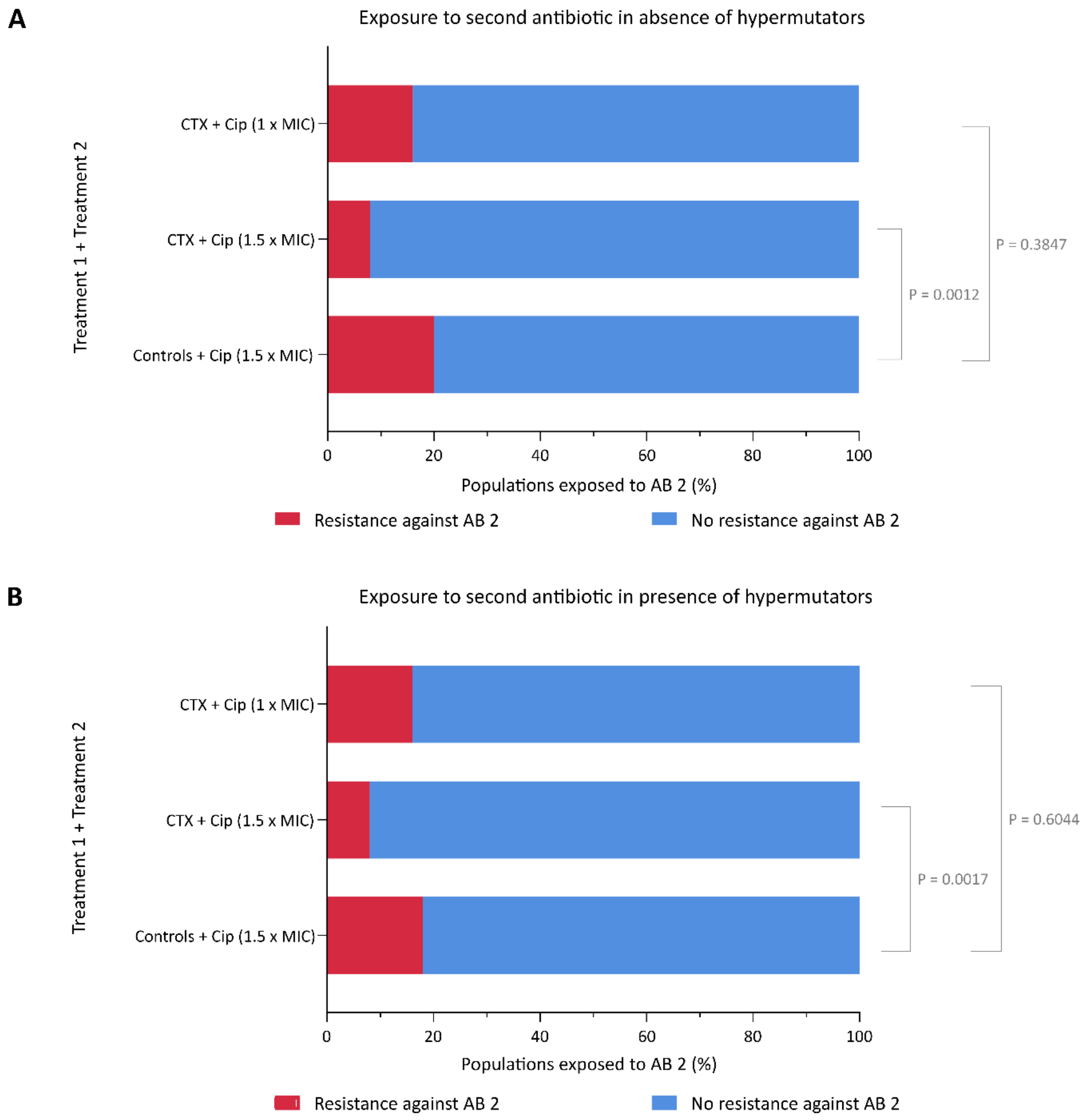

Supplementary Figure S3: Populations that were pre-adapted to cefotaxime prior to evolution against ciprofloxacin showed less adaptation compared to the controls even when the cefotaxime pretreated populations were exposed to lower (1 x MIC) ciprofloxacin concentrations. Percentage of populations able to adapt to ciprofloxacin (Cip; 1.5 x MIC or 1 x MIC) without (A) and with (B) inclusion of hypermutator populations. Increased, but still less, adaptation compared to the controls was observed when cefotaxime pretreated populations were exposed to 1 x MIC instead (16.0 %) or 1.5 x MIC (8.80 %). P-values are derived from a Fisher's Exact test. Source data underlying this figure can be found in Supplementary Data Table S6.

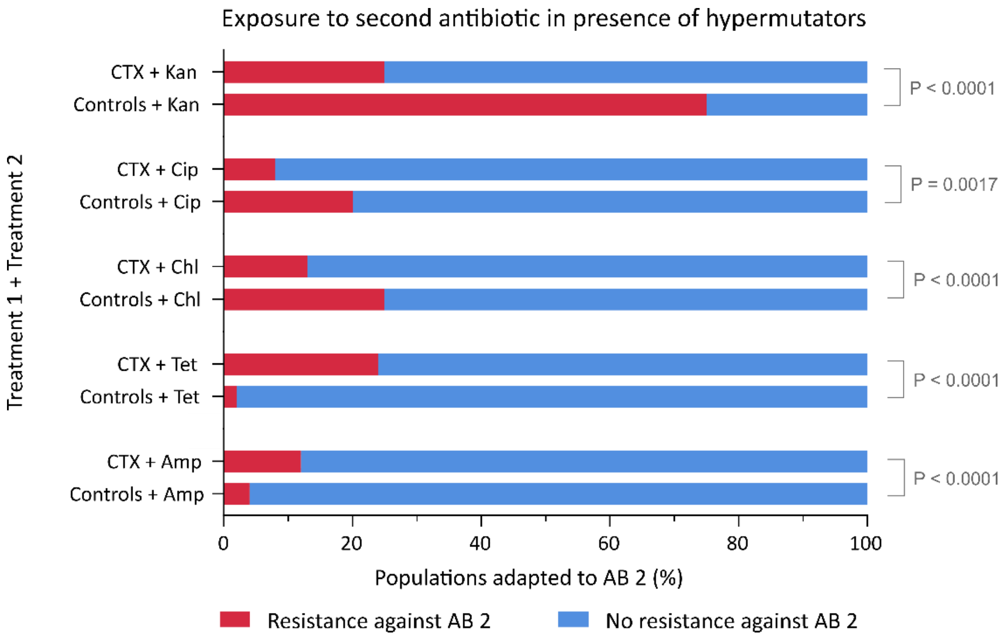

Supplementary Figure S4: Inclusion of hypermutator strains did not impact the adaptation to second antibiotics. Percentage of populations to adapt to a second antibiotic (1.5 x MIC) with inclusion of hypermutator populations. Populations that were pre-adapted to cefotaxime prior to evolution against a second antibiotic show an elevated adaptation compared to the controls when exposed to ampicillin and tetracycline. In contrast, the controls show more adaptation when treated with chloramphenicol, ciprofloxacin and kanamycin. cefotaxime (CTX), ampicillin (Amp), tetracycline (Tet), chloramphenicol (Chl), ciprofloxacin (Cip) and kanamycin (Kan). P-values are derived from a Fisher's Exact test. Source data underlying this figure are provided in Supplementary Data Table S6.

A

Fitness measured in presence of cefotaxime

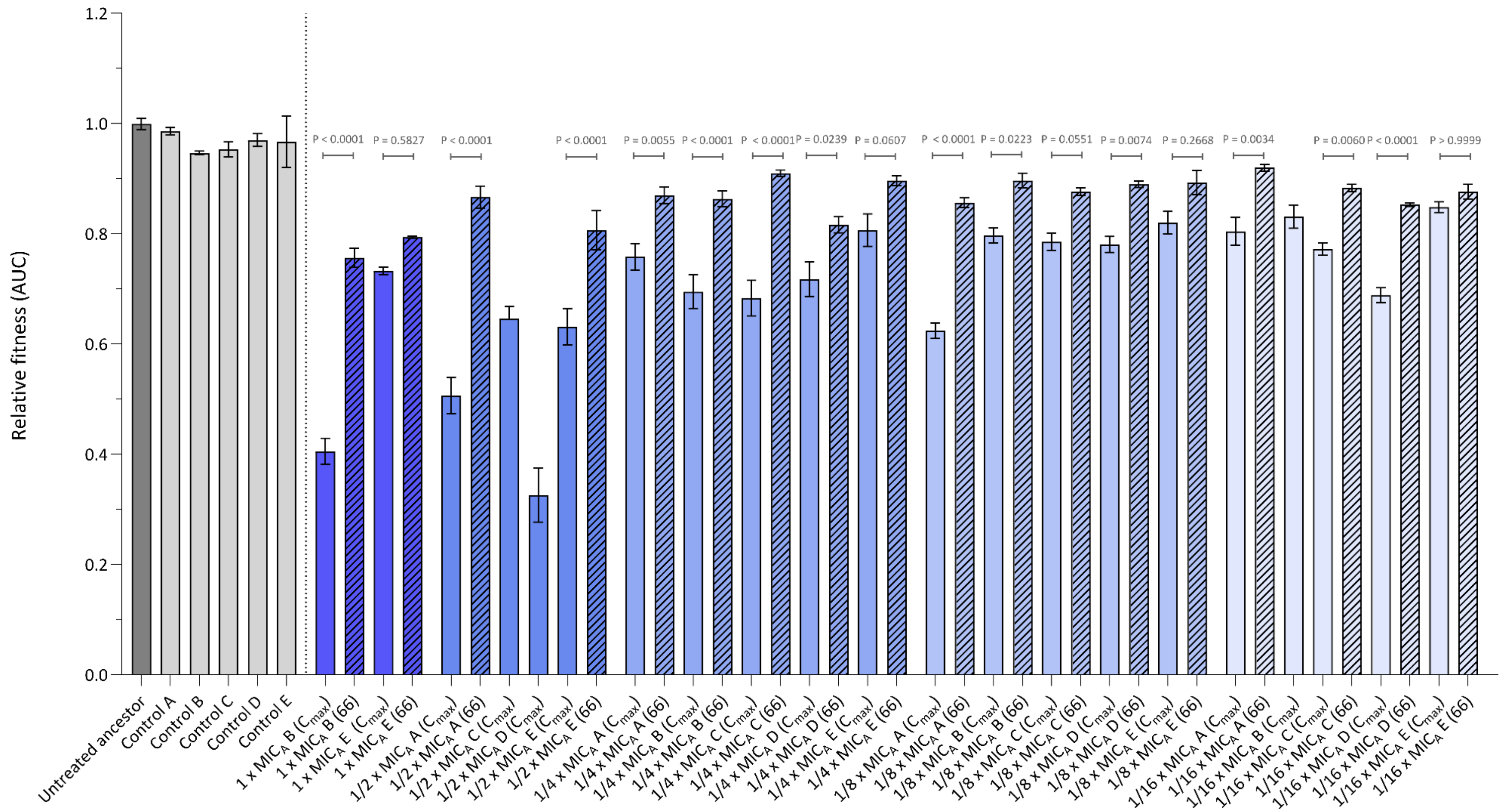

B

Fitness measured in absence of cefotaxime (cost of resistance)

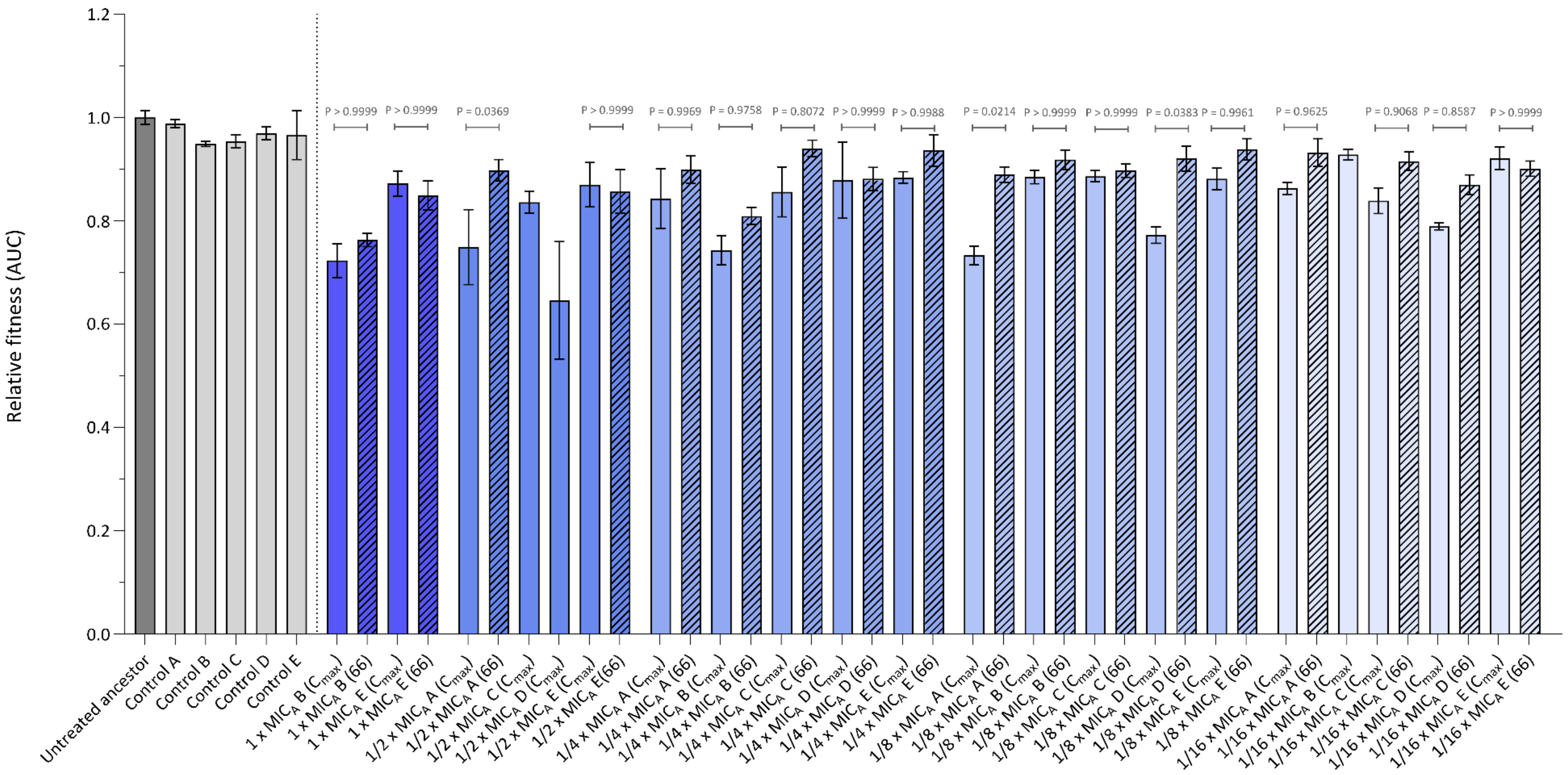

Supplementary Figure S5: Population fitness increases between day C_{max} and day 66 in either the absence or presence cefotaxime. Fitness is measured as the area under the growth curve and expressed relative to an untreated ancestral *S*. Typhimurium strain. Relative fitness increases in both the presence (A) or absence (B) of 4 x MIC_A (0.95 µg.mL^{-1}). cefotaxime. Relative fitness at day C_{max} and day 66 is represented by open and filled bars, respectively. Blue color gradient indicates the applied gradient strength. P-values were derived from One-way ANOVA or Welch ANOV A if S.D were significantly different followed by Sidak's or Dunnet's post hoc test. All P-values can be consulted in Supplementary Data Tables S11 (fitness in presence of cefotaxime) and S12

(fitness in absence of cefotaxime). Error bars denote SEM, n=3. Source data supporting these graphs can be consulted in Supplementary Data Table S11 – Source Data and S12 – Source Data.

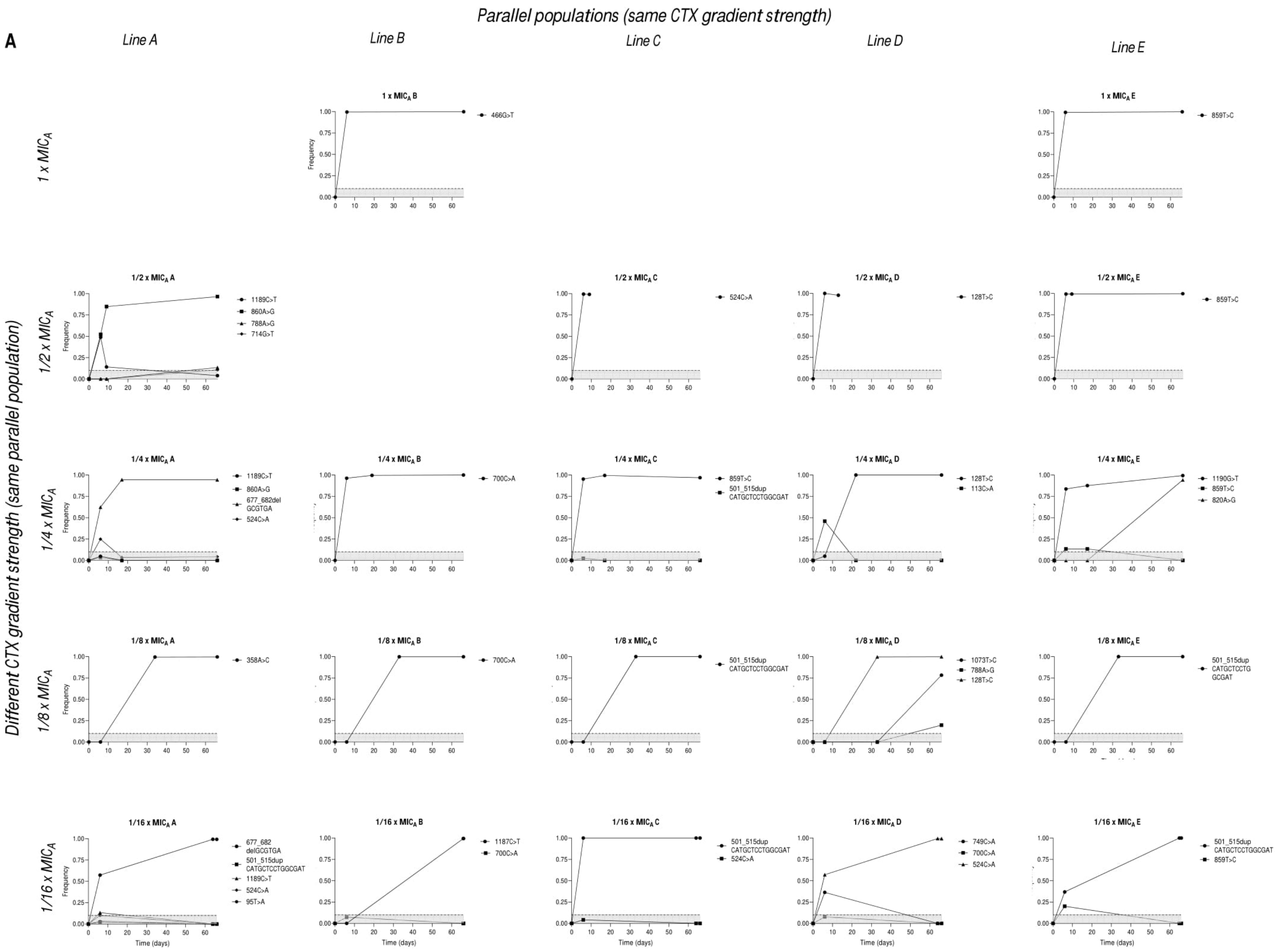

Parallel populations (same CTX gradient strength)
A
Line A
Line B
Line C
Line D
Line E
Different CTX gradient strength (same parallel population)
1 x MIC_A
1/2 x MIC_A
1/4 x MIC_A
1/8 x MIC_A
1/16 x MIC_A
1 x MIC_A B
466G>T
1 x MIC_A E
859T>C
1/2 x MIC_A A
1189C>T
860A>G
788A>G
714G>T
1/2 x MIC_A C
524C>A
1/2 x MIC_A D
128T>C
1/2 x MIC_A E
859T>C
1/4 x MIC_A A
1189C>T
860A>G
677_682del GCGTGA
524C>A
1/4 x MIC_A B
700C>A
1/4 x MIC_A C
859T>C
501_515dup CATGCTCCTGGCGAT
1/4 x MIC_A D
128T>C
113C>A
1/4 x MIC_A E
1190G>T
859T>C
820A>G
1/8 x MIC_A A
358A>C
1/8 x MIC_A B
700C>A
1/8 x MIC_A C
501_515dup CATGCTCCTGGCGAT
1/8 x MIC_A D
1073T>C
788A>G
128T>C
1/8 x MIC_A E
501_515dup CATGCTCCTG GCGAT
1/16 x MIC_A A
677_682 delGCGTGA
501_515dup CATGCTCCTGGCGAT
1189C>T
524C>A
95T>A
1/16 x MIC_A B
1187C>T
700C>A
1/16 x MIC_A C
501_515dup CATGCTCCTGGCGAT
524C>A
1/16 x MIC_A D
749C>A
700C>A
524C>A
1/16 x MIC_A E
501_515dup CATGCTCCTGGCGAT
859T>C
Frequency
Time (days)

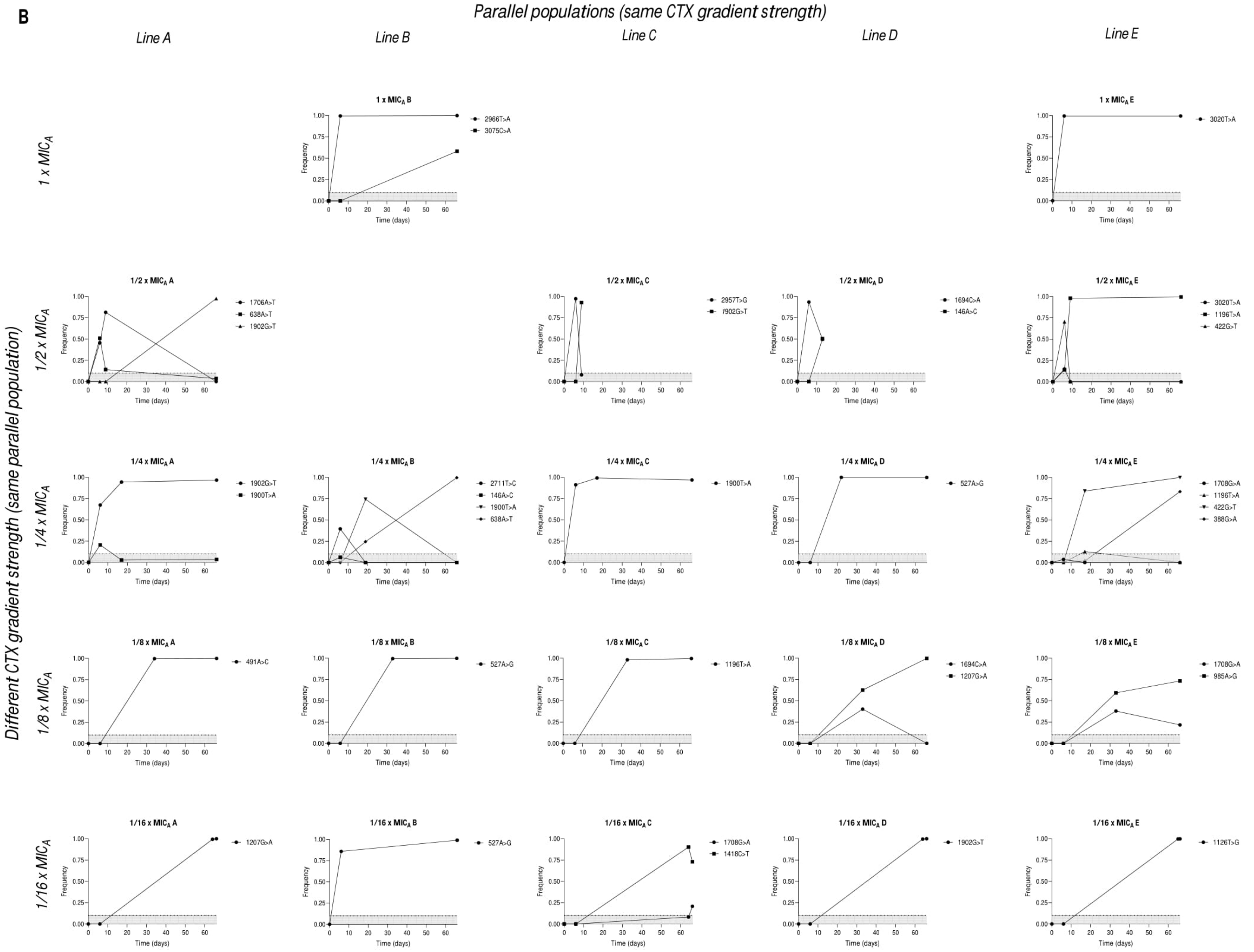
B
Parallel populations (same CTX gradient strength)
Line A
Line B
Line C
Line D
Line E
Different CTX gradient strength (same parallel population)
1 x MIC_A
1/2 x MIC_A
1/4 x MIC_A
1/8 x MIC_A
1/16 x MIC_A
Frequency
Time (days)
1 x MIC_A B
2966T>A
3075C>A
1 x MIC_A E
3020T>A
1/2 x MIC_A A
1706A>T
638A>T
1902G>T
1/2 x MIC_A C
2957T>G
1902G>T
1/2 x MIC_A D
1694C>A
146A>C
1/2 x MIC_A E
3020T>A
1196T>A
422G>T
1/4 x MIC_A A
1902G>T
1900T>A
1/4 x MIC_A B
2711T>C
146A>C
1900T>A
638A>T
1/4 x MIC_A C
1900T>A
1/4 x MIC_A D
527A>G
1/4 x MIC_A E
1708G>A
1196T>A
422G>T
388G>A
1/8 x MIC_A A
491A>C
1/8 x MIC_A B
527A>G
1/8 x MIC_A C
1196T>A
1/8 x MIC_A D
1694C>A
1207G>A
1/8 x MIC_A E
1708G>A
985A>G
1/16 x MIC_A A
1207G>A
1/16 x MIC_A B
527A>G
1/16 x MIC_A C
1708G>A
1418C>T
1/16 x MIC_A D
1902G>T
1/16 x MIC_A E
1126T>G

C

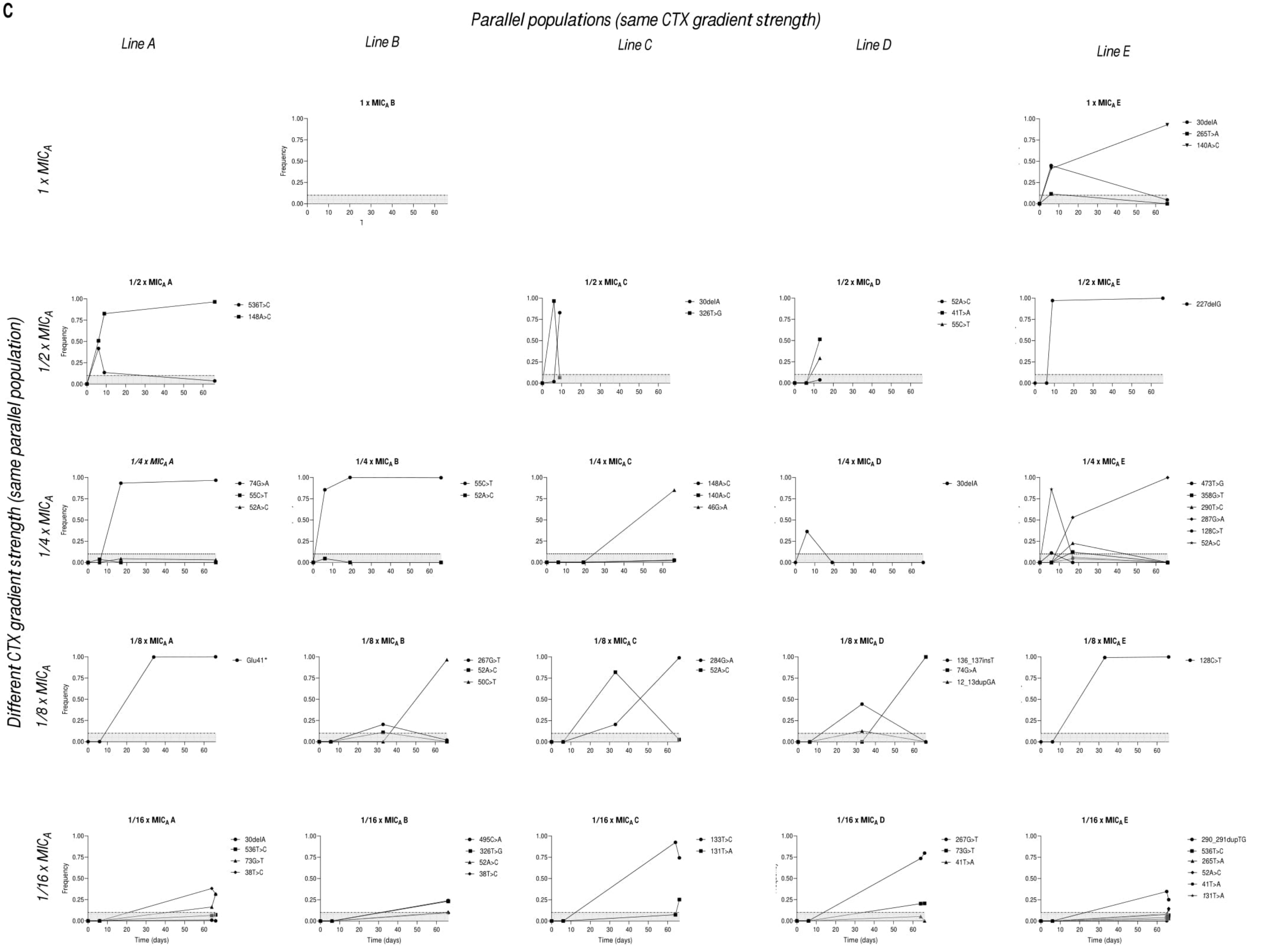

Parallel populations (same CTX gradient strength)
Line A
Line B
Line C
Line D
Line E
Different CTX gradient strength (same parallel population)
1 x MIC$_A$
1/2 x MIC$_A$
1/4 x MIC$_A$
1/8 x MIC$_A$
1/16 x MIC$_A$
1 x MIC$_A$ B
1 x MIC$_A$ E
30delA
265T>A
140A>C
1/2 x MIC$_A$ A
536T>C
148A>C
1/2 x MIC$_A$ C
30delA
326T>G
1/2 x MIC$_A$ D
52A>C
41T>A
55C>T
1/2 x MIC$_A$ E
227delG
1/4 x MIC$_A$ A
74G>A
55C>T
52A>C
1/4 x MIC$_A$ B
55C>T
52A>C
1/4 x MIC$_A$ C
148A>C
140A>C
46G>A
1/4 x MIC$_A$ D
30delA
1/4 x MIC$_A$ E
473T>G
358G>T
290T>C
287G>A
128C>T
52A>C
1/8 x MIC$_A$ A
Glu41*
1/8 x MIC$_A$ B
267G>T
52A>C
50C>T
1/8 x MIC$_A$ C
284G>A
52A>C
1/8 x MIC$_A$ D
136_137insT
74G>A
12_13dupGA
1/8 x MIC$_A$ E
128C>T
1/16 x MIC$_A$ A
30delA
536T>C
73G>T
38T>C
1/16 x MIC$_A$ B
495C>A
326T>G
52A>C
38T>C
1/16 x MIC$_A$ C
133T>C
131T>A
1/16 x MIC$_A$ D
267G>T
73G>T
41T>A
1/16 x MIC$_A$ E
290_291dupTG
536T>C
265T>A
52A>C
41T>A
131T>A
Frequency
Time (days)

D

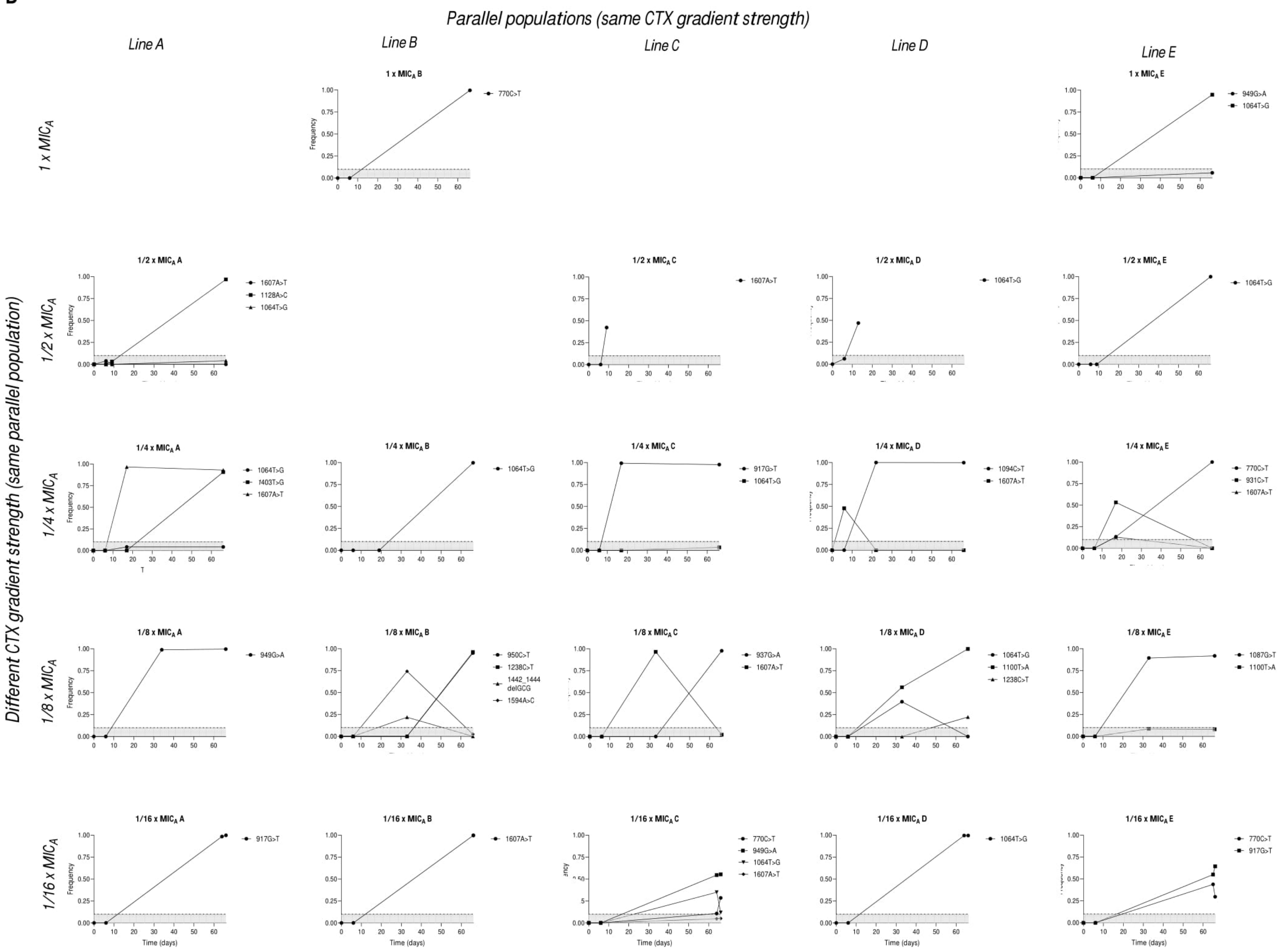
Parallel populations (same CTX gradient strength)
Line A
Line B
Line C
Line D
Line E
Different CTX gradient strength (same parallel population)
1 x MICA
1/2 x MICA
1/4 x MICA
1/8 x MICA
1/16 x MICA
1 x MICA B
770C>T
1 x MICA E
949G>A
1064T>G
1/2 x MICA A
1607A>T
1128A>C
1064T>G
1/2 x MICA C
1607A>T
1/2 x MICA D
1064T>G
1/2 x MICA E
1064T>G
1/4 x MICA A
1064T>G
f403T>G
1607A>T
1/4 x MICA B
1064T>G
1/4 x MICA C
917G>T
1064T>G
1/4 x MICA D
1094C>T
1607A>T
1/4 x MICA E
770C>T
931C>T
1607A>T
1/8 x MICA A
949G>A
1/8 x MICA B
950C>T
1238C>T
1442_1444 delGCG
1594A>C
1/8 x MICA C
937G>A
1607A>T
1/8 x MICA D
1064T>G
1100T>A
1238C>T
1/8 x MICA E
1087G>T
1100T>A
1/16 x MICA A
917G>T
1/16 x MICA B
1607A>T
1/16 x MICA C
770C>T
949G>A
1064T>G
1607A>T
1/16 x MICA D
1064T>G
1/16 x MICA E
770C>T
917G>T
Frequency
Time (days)

Supplementary Figure S6: Cefotaxime treated populations accumulated resistance mutations in four key driver genes. Evolutionary dynamics of mutations in the four key driver genes of cefotaxime resistance namely *envZ* (A), *acrB* (B), *ramR* (C) and *ftsI* (D). Populations were sequenced at three different time points namely, day 6 – day C_{max} – and day 66. Source data underlying these figures can be found in Supplementary Data Tables S15 - S16 - S17 - S18.

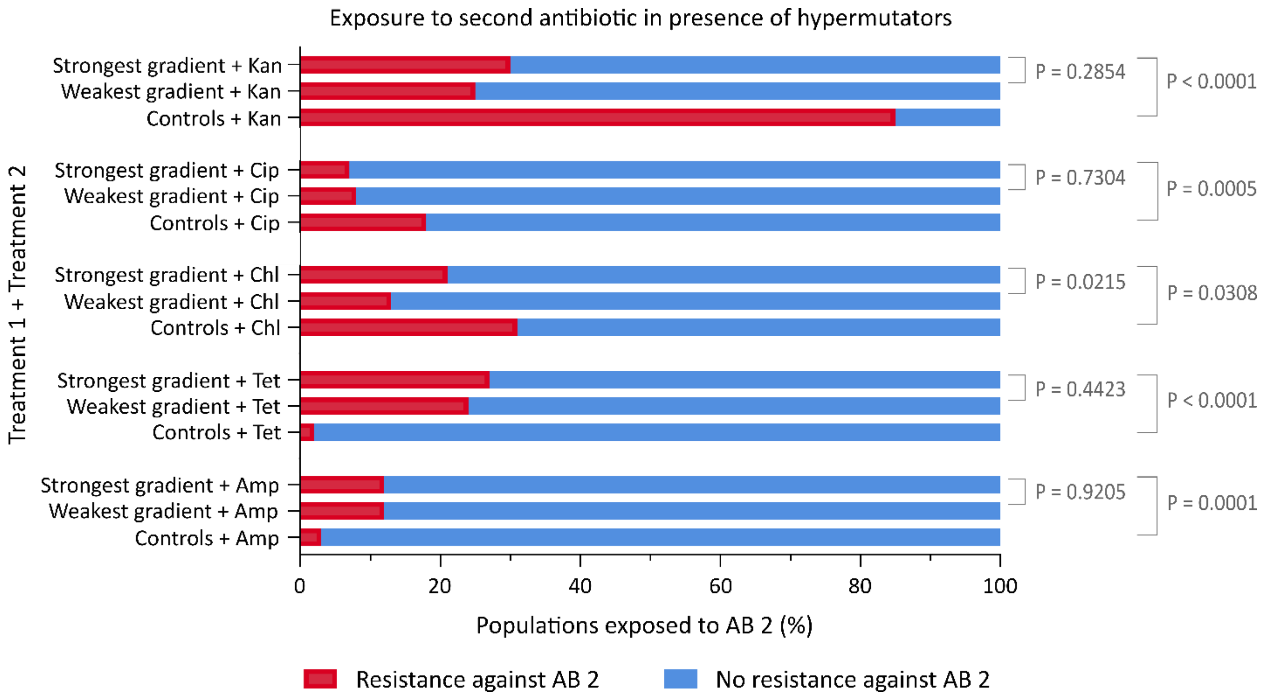

Supplementary Figure S7: **Inclusion of hypermutator strains does not result in a signficant difference between the strongest and weakes gradients except for chloramphenicol.** Percentage of populations able to adapt to a second antibiotic (1.5 x MIC). When hypermutator populations were included, no significant difference in adaptation between the strongest and weakest cefotaxime gradients was observed, except for chloramphenicol. Populations pre-adapted to a strong cefotaxime gradient however tend to show more adaptation compared to populations exposed to the weakest cefotaxime gradients when exposed to kanamycin and tetracycline. In contrast, the weakest gradient strengths tended to show more adaptation compared to the strongest gradients when ciprofloxacin was applied. No difference was found in case of ampicillin. ampicillin (Amp), tetracycline (Tet), chloramphenicol (Chl), ciprofloxacin (Cip) and kanamycin (Kan). P-values are derived from a Fisher's Exact test. Source data are provide in Supplementary Data Table S6.

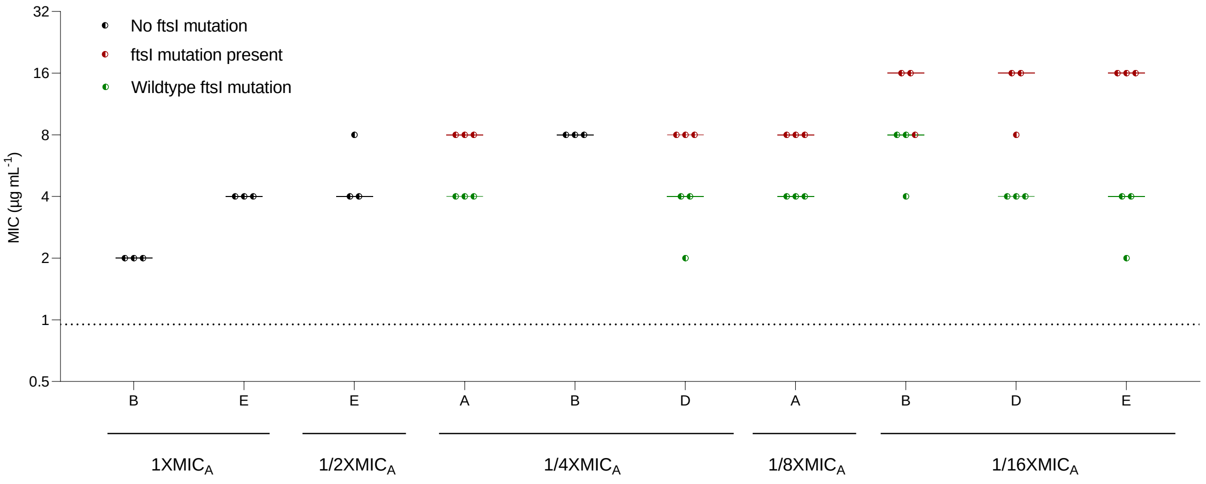

Supplementary Figure S8: *ftsI* mutations are drivers for high cefotaxime resistance. Cefotaxime resistance decreased when *ftsI* mutations were reverted to the background of an untreated ancestral *S.* Typhimurium and reached similar resistance levels as populations that did not accumulate a mutation in *ftsI*. Each dot represents the MIC of one biological repeat (n=3). C_{max} and the median MIC of three repeats (n=3) are indicated by the dashed line and horizontal lines, respectively.

Bibliography

Jahn LJ, Munck C, Ellabaan MMH, Sommer MOA. 2017. Adaptive Laboratory Evolution of Antibiotic Resistance Using Different Selection Regimes Lead to Similar Phenotypes and Genotypes. Front Microbiol. 8:816.

Sun S, Berg OG, Roth JR, Andersson DI. 2009. Contribution of gene amplification to evolution of increased antibiotic resistance in Salmonella typhimurium. Genetics. 182(4):1183–1195.

Sun S, Selmer M, Andersson DI. 2014. Resistance to β-lactam antibiotics conferred by point mutations in penicillin-binding proteins PBP3, PBP4 and PBP6 in Salmonella enterica. PLoS One. 9(5):1–10.
